# Cardiopulmonary progenitors facilitate cardiac repair via exosomal transfer of miR‐27b‐3p targeting the SIK1‐CREB1 axis

**DOI:** 10.1111/cpr.13593

**Published:** 2024-01-07

**Authors:** Ying‐Ying Xiao, Luo‐Xing Xia, Wen‐Jing Jiang, Jian‐Feng Qin, Li‐Xin Zhao, Zhan Li, Li‐Juan Huang, Ke‐Xin Li, Peng‐Jiu Yu, Li Wei, Xue‐Yan Jiang, Zhe‐Sheng Chen, Xi‐Yong Yu

**Affiliations:** ^1^ Key Laboratory of Molecular Target & Clinical Pharmacology, the NMPA and State Key Laboratory of Respiratory Disease, School of Pharmaceutical Sciences, The Fifth Affiliated Hospital & the First Affiliated Hospital Guangzhou Medical University Guangzhou Guangdong China; ^2^ Department of Pharmacy, The First Affiliated Hospital Guangzhou Medical University Guangzhou Guangdong China; ^3^ Department of Pharmaceutical Sciences, College of Pharmacy and Health Sciences, Institute for Biotechnology St. John's University Queens New York USA

## Abstract

Ischemic heart disease, especially myocardial infarction (MI), is one of the leading causes of death worldwide, and desperately needs effective treatments, such as cell therapy. Cardiopulmonary progenitors (CPPs) are stem cells for both heart and lung, but their repairing role in damaged heart is still unknown. Here, we obtained CPPs from E9.5 mouse embryos, maintained their stemness while expanding, and identified their characteristics by scRNA‐seq, flow cytometry, quantitative reverse transcription‐polymerase chain reaction, and differentiation assays. Moreover, we employed mouse MI model to investigate whether CPPs could repair the injured heart. Our data identified that CPPs exhibit hybrid fibroblastic, endothelial, and mesenchymal state, and they could differentiate into cell lineages within the cardiopulmonary system. Moreover, intramyocardial injection of CPPs improves cardiac function through CPPs exosomes (CPPs‐Exo) by promotion of cardiomyocytic proliferation and vascularization. To uncover the underlying mechanism, we used miRNA‐seq, bulk RNA‐seq, and bioinformatic approaches, and found the highly expressed miR‐27b‐3p in CPPs‐Exo and its target gene *Sik1*, which can influence the transcriptional activity of CREB1. Therefore, we postulate that CPPs facilitate cardiac repair partially through the SIK1‐CREB1 axis via exosomal miR‐27b‐3p. Our study offers a novel insight into the role of CPPs‐Exo in heart repair and highlights the potential of CPPs‐Exo as a promising therapeutic strategy for MI.

## Graphical abstract

CPPs improve recovery from cardiac injury in mice by their exosomes through the promotion of cardiomyocytic proliferation and vascularization partially by miR‐27b‐3p mediating the SIK1‐CREB1 axis.

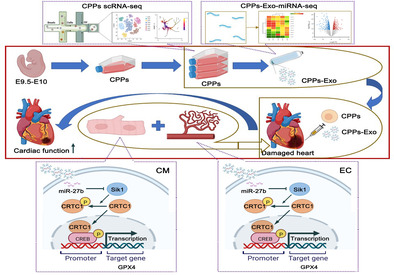

## 1 INTRODUCTION

Ischemic heart disease, particularly myocardial infarction (MI), characterized by massive cardiomyocyte (CM) death followed by cardiac dysfunction and myocardial fibrosis, is one of the leading causes of death on a global scale^1^ and causes enormous social stress and economic burden.^2^ The majority of cells lost in MI are contractile CMs, which could be up to 1 billion. Unfortunately, the extremely low proliferative rate of CMs results in the replacement of dead cells with a fibrotic scar, which protects the damaged myocardial wall from rupture, impairs proper cardiac contraction, and eventually ends in heart failure (HF). In addition to CMs, other types of cells, such as smooth muscle cells and endothelial cells (EC), also suffer collateral damage, and their functional renewal is essential for effective heart repair.^3^ Currently, conventional therapies aim to reoxygenate the infarct myocardium by interventions such as coronary artery bypass surgery or percutaneous coronary intervention to prevent enlargement of the infarction area and HF.^4^ Due to the rare proliferative potential of mature CMs, it is imperative to find a new way to replenish the lost cells in the infarcted heart. Nowadays, accumulated evidence has shown that cell therapy holds great potential for cell regeneration.

Since intrinsic repair mechanisms are insufficient to restore cardiac function after injury, the focus turns to other approaches, which include delivering various cell populations, for example, stem/progenitor cells such as human embryonic stem cells (hESCs) and human‐induced pluripotent stem cells,^5^, ^6^ and their derived cardiovascular cells, such as cardiac progenitor cells (CPCs), hESC‐derived cardiovascular progenitor cells^7^ and cardiosphere‐derived cells (CDC).^8^ Recently, several Phase II/III, double‐blind, randomized, multi‐centre clinical trials have obtained remarkable effects, including ixCELL‐DCM,^9^ MSC‐HF,^10^ CONCERT‐HF,^11^ DREAM‐HF,^12^, ^13^ and TAC‐HFT.^14^ Strikingly, the treatment was given only once, and the outstanding outcome was measured 12 months later,^15^ implying that cell therapy has tremendous potential. However, the ideal cells are still under exploration and should be able to differentiate into a variety of key cardiac lineage cells (e.g., CMs and EC) while proliferating in vitro and integrating effectively with host cells.^16^

Cardiopulmonary progenitor cells (CPPs) that contribute to both heart and lungs development are a recent addition to cell populations generating the heart.^17^ However, virtually nothing is known about their contribution to MI. CPPs originate from the cardiac inflow tube and ventral side of the anterior vestgut and specifically express the typical Wnt signalling pathway ligand Wnt2, the Hedgehog (Hh) signalling pathway response transcription factor Gli1, and the LIM/homeodomain family transcription factor Isl1.^17^ Unlike other CPCs, CPPs have the ability to differentiate into cells belonging to dual lineages (heart and lung lineages),^17^ and produce atrial septum and cardiorespirovascular system.^18^ As a heterogeneous pool of progenitor cells, CPPs can differentiate into either myocardial cells that are lost mostly during MI, or EC and smooth muscle cells, which are collateral damage caused by MI, suggesting that CPPs hold tremendous promise for cardiac regeneration. Nonetheless, there is no reported research on whether CPPs have the ability to repair the heart. Articles on CPPs are few and focus mainly on the regulation of heart or lung development.^19^ The roles of CPPs in repairing damaged hearts need to be investigated.

The mechanisms underlying the promotion of heart regeneration and the repair of cardiac function by cell therapy are controversial. One opinion is that transplanted stem cells or their derivatives directly differentiate into mature CMs and vascular cells (e.g., smooth muscle cells and EC) after implantation in damaged host heart tissue; the other is that stem cells or their derivatives promote the proliferation of original CMs in the damaged heart through paracrine factors and induce angiogenesis in ischemic areas.^20^ Data from several clinical trials of stem cells or their derivatives (such as SCIPIO‐NCT00474461, CADUCEUS‐NCT00893360, and CAREMI‐NCT02439398) have shown that although the retention rate of cells after transplantation was extremely low, some physiological improvements could still be achieved after treatment, implying that cell transplantation was effective critically via paracrine mechanisms, rather than through differentiation and functional integration of transplanted cells.^21^

Key players in paracrine signalling are extracellular vesicles (EVs), including exosomes and particles.^22^ Among them, exosomes are vesicles with an inner diameter of 50–150 nm and are formed by the inward germination of the endoplasmic membrane.^23^ Exosomes can encapsulate different biomolecules, for instance, lipids, proteins, and nucleic acids, such as non‐coding RNAs, among which, microRNAs (miRNAs) have attracted the most attention for their high conservation across species^24^ and powerful regulatory roles in gene expression,^25^ while lncRNAs and circRNAs were less well studied and their mechanism of action often remains obscure. Moreover, the therapeutic potential of miRNAs is becoming clear, while the potential of lncRNAs and circRNAs for cardiac regeneration in humans remains to be determined. Because small rodent models are the primary in vivo tools for cardiac regeneration research, the safety and efficacy of novel ncRNA therapeutics based on lncRNAs and circRNAs will require evaluation in large animals prior to the initiation of human clinical trials.^26^ Therefore, we select exosomal miRNA as the primary focus in our investigation.

The miRNAs in EVs derived from various types of cells, such as mesenchymal stem cells (MSCs),^27^, ^28^ Sca1+ adult cells,^29^ CDC^30^ and mouse ESCs,^31^ have been shown to protect the heart from MI injury. As an epigenetic regulator of genes, miRNAs have become an important direction for decoding the mechanisms underlying treatment for regenerative repair after heart injury. In our study, we examined the efficacy of CPPs exosomes (CPPs‐Exo) in heart repair, and the role of specific miRNAs derived from CPPs‐Exo during this process.

Collectively, in this study, we aim to discuss the potential of CPPs in repairing the heart after MI, with three major scientific questions that need to be resolved: (i) whether CPPs could repair damaged hearts caused by MI; (ii) how CPPs could restore heart function by their own abilities of proliferation and differentiation, or through their exosome‐mediated paracrine pathways; and (iii) whether miRNAs in CPPs‐Exo exert beneficial effects on heart function through epigenetic regulation, which enables the regulation of target genes related in CM proliferation and angiogenesis. Our findings have isolated and expanded CPPs and reveal that CPPs are able to improve heart function after MI through the epigenetic regulation of CPP‐Exo, all of which provide a new CPC population and scientific basis for the treatment of patients with ischemic heart disease.

## 2 MATERIALS AND METHODS

### 2.1 Mice

C57BL/6 mice were obtained from GemPharmatech™. All procedures in mice were approved by the Institutional Animal Care and Use Committee of Guangzhou Medical University (Acceptance number: G2021‐010) and were conformed to the guidelines from Directive 2010/63/EU of the European Parliament on the protection of animals used for scientific purposes. Mice were housed on a 12/12‐h light/dark cycle and fed and watered ad libitum. At the end of the experiment, mice were sacrificed by cervical dislocation after an overdose of isoflurane (dose 5%).

### 2.2 Embryo preparation

Timed matings were set up between male (8–10 weeks of age) and female (6–8 weeks of age) mice. Noon on the day of the vaginal plug was considered as embryonic day (E) 0.5. Pregnant female mice were sacrificed by cervical dislocation after an overdose of isoflurane (dose 5%) to harvest embryos at E9.5 for scRNA‐seq and CPPs isolation.

### 2.3 CPPs isolation and culture

Under a dissecting microscope, the head and tail of the C57BL/6 mouse embryos at E9.5 were removed, and then the CPPs region was separated and digested with digestion solution (0.04% trypsin and 0.05% collagenase IV) at 37°C for 10 min. The collected cells were cultured in suspension drops by the embryonic bodies method for 2 days, digested into a single‐cell suspension, and cultured 1 day with differentiation medium (RPMI 1640 with 2% B‐27 without insulin, 2 mM L‐glutamine, 1% NEAA, 1% penicillin/streptomycin, 0.1 mM β‐mercaptoethanol) supplemented with 12 μM CHIR99021, followed by differentiation medium without CHIR99021 for another day. The cells were then digested into a single‐cell suspension, plated on a Petri dish coated with 0.2% gelatin gum, and cultured with ABC medium (DMEM/F12 supplemented with 2% B‐27 without vitamin A, 2 mM L‐glutamine, 1% nonessential amino acids, 0.1 mM beta‐mercaptoethanol, 2.5% human platelet lysate, 0.5 μM A83‐01, 50 ng/mL basic fibroblast growth factor [bFGF], and 12 μM CHIR‐99021). The medium was changed every 2 days. CPPs were not passaged until the cell confluency was >90%.

### 2.4 Flow cytometry

Flow cytometry analyses were performed with a flow cytometer (BD Biosciences) and FlowJo V10 software. Briefly, cells were blocked with 5% bovine serum albumin (BSA) and stained with a panel of antibodies for 1 h at room temperature with or without sequential incubation with secondary antibodies. The expression of antigen markers was analysed by flow cytometry detection. The antibodies used can be found in Table S1.

### 2.5 Single‐cell sequencing

For library construction and sequencing, cellular suspensions were loaded on a 10× Genomics GemCode Single‐cell instrument that generates single‐cell Gel Bead‐In‐EMlusion (GEMs). Libraries were generated and sequenced from the cDNAs with Chromium Next GEM Single Cell 3′ Reagent Kits v3.1. Upon dissolution of the Gel Bead in a GEM, primers containing (i) an Illumina® R1 sequence (read 1 sequencing primer), (ii) a 16 nt 10× Barcode, (iii) a 10 nt Unique Molecular Identifier (UMI), and (iv) a poly‐dT primer sequence were released and mixed with cell lysate and Master Mix. Barcoded, full‐length cDNAs were then reverse‐transcribed from polyadenylated mRNA. Silane magnetic beads were used to remove leftover biochemical reagents and primers from the post‐GEM reaction mixture. Full‐length, barcoded cDNAs were then amplified by polymerase chain reaction (PCR) to generate sufficient mass for library construction. Sequencing was performed on an Illumina Novaseq 6000 sequencer using a pair‐end 150 bp (PE150) reading strategy (performed by Gene Denovo Biotechnology Co, Guangzhou, China).

For clustering analysis, alignment, filtering, barcode counting, and UMI counting were performed with Cell Ranger to generate a feature‐barcode matrix and their global gene expression. Dimensionality reduction, visualization, and analysis of the scRNA sequencing data were performed with the R package Seurat.

(version 3.1.2). As a further quality‐control measure, cell that met any of the following criteria were filtered out: <360 or >5400 unique genes expressed, >52,000 UMIs, or >30% of reads mapped to the mitochondria. After removing unwanted nuclei from the datasets, 2000 highly variable genes were used for the downstream clustering analysis. Principal component analysis was performed, and the cell types and subtypes were annotated according to their expression of the known canonical marker genes of the respective cell types. Cell sub‐clusters with similar gene expression patterns were annotated as the same cell type.

For differentially expressed genes (DEGs) analysis, the expression level of each gene in the target cluster was compared with other cells using the Wilcoxon rank‐sum test. The significantly up‐regulated genes were identified using the following criteria: (1) at least 1.28‐fold overexpression in the target cluster, (2) expression in more than 25% of the cells belonging to the target cluster, and (3) fause discovery rate (FDR) is less than 0.05.

For gene ontology (GO) and Kyoto encyclopedia of genes and genomes (KEGG) pathway enrichment analysis, the DEGs in CPPs cluster comparing with the other five cell clusters were first identified, and then located in cellular processes and pathways. The calculated *p*‐value was gone through FDR correction, taking FDR ≤0.05 as a threshold. GO term and KEGG pathways that met this condition were defined as the significantly enriched pathways in DEGs.

For pseudotime analysis, single‐cell trajectory analysis was applied using two methods. The first one is Monocle (http://cole-trapnell-lab.github.io/monocle-release/) with DDRTree and default parameters. Prior to Monocle analysis, marker genes of the Seurat clustering result and raw expression counts of the cells that passed filtering were selected. Based on pseudotime analysis, branch expression analysis modelling was applied for branch fate‐determined gene analysis. The other method, CytoTRACE (https://cytotrace.stanford.edu/) was used to determine differentiation potential.

### 2.6 Karyotyping

When the cell confluence reached 60%, colchicine was added at a concentration of 100 ng/mL at 37°C for 1.5 h. Then the cells were digested, centrifuged, and washed once with phosphate‐buffered saline (PBS) before being re‐suspended in hypotonic KCl at 37°C for 20–40 min. After fixation, the supernatant was discarded the next day, and the cells were dropped on pre‐cooled slides immediately, dried for 1–2 h, and then digested by pancreatic enzymes. The digestion was stopped by saline, and the cells were stained with Giemsa staining solution, and dried at room temperature. After GTG‐banding, 10 metaphase spreads were analysed for each sample using CytoVision software v.7.0 (Leica).

### 2.7 Extraction and analysis of CPPs‐Exo

Exosomes secreted by CPPs were isolated by ultracentrifugation. Briefly, the culture medium was collected and centrifuged at 300 g for 10 min, followed by centrifugation at 2000 g for 10 min to remove cell debris, and then centrifuged at 10,000 g for 30 min prior to centrifugation for 70 min at 100,000 g. Finally, the pellets were re‐suspended in PBS, and aliquoted at −80°C for storage. We determined the concentration of exosomes by the BCA method, detected the morphology of exosomes with transmission electron microscopy (TEM), measured the exosomes particle size using nanoparticle tracking analysis (NTA), and identified the surface markers of exosome, such as CD63, Alix, and Hsp70 by means of Western blot.

### 2.8 Establishment of a mouse acute MI model and intramyocardial PBS/CPPs/CPPs‐Exo delivery

Female (7–9 weeks of age) C57BL/6 mice were anaesthetised with an intraperitoneal injection of 0.02 mL/g 1.25% Afodin (Easycheck). The mice were orally intubated with a 24‐gauge tube and mechanically ventilated at 125 breaths/min with a tidal volume of 120 μL. The thorax was opened by a lateral thoracotomy, and the heart was exposed by a pericardial incision. An 8‐0 nylon suture was placed under the left anterior descending inferior artery (LAD) for permanent ligation, which was verified by evident paling of the left ventricle below the stitch site. Immediately after coronary occlusion, the mice were injected intramyocardially with 15 μL of PBS, CPPs (0.4 million cells/heart), or CPPs‐Exo (50 μg, 15 μL) at three sites of the infarct border zone. The chest was closed, and the mice were allowed to recover.

Animals were sacrificed by cervical dislocation after an overdose of isoflurane (dose 5%) for tissue harvesting and histological assay 6 weeks after LAD ligation.

### 2.9 Histology

For cellular immunohistochemistry, briefly, cells were plated on confocal dishes (Millipore) and fixed with 4% paraformaldehyde. After permeabilization with 0.5% Triton X‐100 and blocking with 5% BSA, the cells were incubated with the primary antibody at 4°C overnight, and then incubated with the fluorescent secondary antibody at room temperature, followed by counterstaining the nuclei with 4,6‐diamidino‐2‐phenylindole (DAPI). The primary and secondary antibodies can be found in Table S1.

For tissue immunohistochemistry, briefly, mouse hearts were fixed with 10% formalin followed by 30% sucrose, frozen in optimum cutting temperature compound and processed for sectioning. We performed heat‐induced epitope retrieval in 10 mM citrate buffer (pH 6.0) followed by 5% BSA blocking and staining with primary antibodies overnight at 4°C followed by incubation with secondary antibodies before counterstaining the nuclei with DAPI. The primary and secondary antibodies can be found in Table S1. Staining was analysed by a Zeiss 780 laser scanning microscope (Carl Zeiss). The number of capillaries and vessels was calculated manually and expressed as vessel density per 1 mm^2^. Proliferative CMs were classified as cardiac troponin T (cTnT) positive cells with Ki67‐positive nuclei in each field.

Masson staining was performed using a commercial kit according to the manufacturer's instructions (Millipore, Sigma). The thickness of infarct wall was assayed by Image J.

For Sirius red staining, paraffin sections were dewaxed, hydrated by gradient ethanol, and stained by lapis lazuli blue for 5–10 min. After rinsing with distilled water three times, the sections were stained with Sirius red saturated bitter acid solution for 15–30 min, differentiated by anhydrous ethanol, dehydrated, and sealed by xylene transparent optical gum before scanning and photography.

For HE staining, heart cryosections were fixed in haematoxylin for 5 min at room temperature and then rinsed in tap water for 2 min. The slices were then soaked in acidic alcohol, sodium bicarbonate, and dehydrating solution sequentially. Finally, they were immersed in Akebono for 2 min and thoroughly washed in dehydrating agent and xylene.

### 2.10 Echocardiography

Echocardiographic studies were performed using a Vevo 2100 imaging system (VisualSonics). Briefly, the mice were anaesthetised with 2% isoflurane inhalation and the heart rate was maintained at 400–500 beats/min. The M‐mode image of the left ventricular short axis was used to measure the left ventricular end‐systolic volume (LVESV) and end‐diastolic volume (LVEDV). Left ventricular ejection fraction (EF) was calculated as [LVEDV − LVESV]/LVEDV, and left ventricular fractional shortening (FS) was calculated as [LVIDd − LVIDs]/LVIDd. Digital images were analysed offline by blind observers using Vevo 2100 Workstation 1.7.1.

### 2.11 Quantitative reverse transcription‐PCR detection

Total RNA was isolated using TRIzol reagent (Invitrogen). Quantitative reverse transcription‐PCR (RT‐qPCR) was performed by using SYBR Green PCR Master Mix (Takara) in a LightCycler 480 System (Roche). The primers are listed in Table S2.

### 2.12 Western blot

Exosomes or cells were lysed in RIPA buffer with Triton X‐100 before quantification by the BCA method. A 20 μg protein sample was loaded, electrophoresed by 10% sodium dodecyl sulfate polyacrylamide gel electrophores (SDS‐PAGE), transferred to a polyvinylidene difluoride (PVDF) membrane (Millipore) at 4°C for 2 h at 100 mA, and placed in blocking solution at 37°C for 1 h before exposure to the primary antibody overnight at 4°C. The next day, after washing, the membrane was exposed to the secondary antibody for 1 h at room temperature. Then the membrane was washed before the colour was developed. The results were scanned using an Amersham Imager 600 (GE). The primary and secondary antibodies used can be found in Table S1.

### 2.13 miRNA‐seq analysis

Small RNA‐sequencing libraries were prepared using the TruSeq Small RNA Sample Prep Kit (Illumina) kit. After the library preparation was completed, the constructed library was sequenced using Illumina Hiseq 2000/2500, with a single‐ended reading length of 50 bp. The analysis process was as follows: clean reads were obtained after quality control processing with the original data. The clean reads were removed from 3′ joints, and length screening was performed, retaining the sequence of base lengths of 18–26 nt. The remaining sequences were then compared with various RNA database sequences (excluding miRNA), such as the mRNA database, RFam database (containing rRNA, tRNA, snRNA, snoRNA, etc.), and Repbase database (repeat sequence database), filtered, and finally, the final data obtained were valid data, which could be used for subsequent small RNA data analysis.

### 2.14 Cell counting kit‐8 (CCK‐8) assay

Cells were seeded in a 96‐well plate. After 1 day of starvation culture with serum‐free medium, cells were treated with exosomes or miRNAs. CCK‐8 was added to the well 1.5 h before detecting the absorbance value at 450 nm with a UV spectrophotometer.

### 2.15 Scratch assay

Cells were plated in a 6‐well plate, and the medium was changed to serum‐free medium the next day overnight. Crosses were drawn with the tip of the pipette before replacement of the medium with complete medium supplemented with exosomes or miRNAs. The scratches were photographed immediately and every 24 h.

### 2.16 Transwell invasion assay

The cells were plated on a Transwell plate, switched to serum‐free medium the next day, and exosomes or miRNAs were added. The next day, the cells were fixed with 4% paraformaldehyde for 10 min, and then stained with 0.1% crystal violet for 10 min. The cells on the bottom side of the Transwell chamber were smeared with a cotton swab, and the chamber was washed with distilled water, dried, and imaged.

### 2.17 Tube formation assay

Starved EC were seeded in a 96‐well plate coated with Matrigel (1:3 dilution), and different treatments, such as exosomes and miRNAs, were added 1 h later. Photographs were taken after 4–6 h.

### 2.18 Teratoma formation assay

A total of 5 million CPPs or mESCs (cells resuspended in Matrigel and medium in 1:1) were injected on the right side of male immunodeficient mice at 8 weeks old. The growth of teratomas was monitored weekly for a period of 90 days.

### 2.19 Bulk‐RNA sequencing analysis

The collected total cell RNA was extracted using the QIAGEN RNA extraction kit, according to the manufacturer's instructions. The ribosomal RNA in the cell was removed, and the RNA was broken into a fragment of 200 bp size before synthesizing the cDNA followed by the construction of the library and sequencing.

### 2.20 Dual‐luciferase reporter assay

The 3′ untranslated regions (3′UTR) regions, including wild‐type miRNA binding sites, and the mutated 3′UTR were cloned downstream of the firefly luciferase gene within the psiCHECK‐2 vector (Promega). For reporter assays, HEK‐293T cells were seeded in 96‐well plates in triplicate and allowed to settle for 24 h. The cells were then co‐transfected with 10 ng firefly luciferase reporter plasmid and an equal amount (200 nM) of miR‐130b‐3p mimic or scrambled negative control RNA using Lipofectamine 2000 (Invitrogen). At 24 h post‐transfection, the cells were assayed using a luciferase assay kit (Promega).

### 2.21 Nanoparticle tracking analysis

The size of exosomes was determined by NTA using a NanoSight LM 10 instrument (Malvern) equipped with Viton sample room and laser (640 nm). Exosomes were re‐suspended in PBS and then diluted with Milli‐Q water 500 times, followed by injection into sample chamber using a sterile syringe. The granularity value assessed by the NTA software corresponds to the arithmetic value of all particle sizes analysed by the software.

### 2.22 Transmission electron microscopy

To characterize the exosomes, 30 μL of exosomes were stained in 30 μL of phosphotungstic acid solution (pH = 6.8). Next, exosomes were analysed using TEM.

### 2.23 Chromatin immunoprecipitation‐qPCR

HMEC‐1 cells were transfected with si‐Sik1 for 72 h and subjected to chromatin immunoprecipitation (ChIP) by using a Magna ChIP A/G Chromatin Immunoprecipitation Kit (Sigma‐Aldrich) according to the manufacturer's manuals. Briefly, the cells were fixed with 1% formaldehyde, washed with ice‐cold PBS containing protease inhibitor cocktail, and then lysed with lysis buffer containing protease inhibitor cocktail. After sonication to break DNA to 200–450 bp, anti‐CREB antibody (Table S1) or normal mouse IgG and protein A/G magnetic beads were added to the supernatant and incubated for 1 h at 4°C with rotation. The Protein A/G magnetic bead‐antibody/chromatin complex was extensively washed and eluted with elution buffer with proteinase K. The total DNA was purified via phenol/chloroform extraction and ethanol precipitation, and subjected to qPCR. The primers flanking the CREB binding site of the *Gpx4* promoter are listed in Table S2.

### 2.24 Statistical analysis

The results are presented as the mean ± standard error of the mean (SEM). An unpaired two‐sided *t*‐test was used for comparisons between two different groups. A value of *p* < 0.05 was considered statistically significant (ns *p* > 0.05, * *p* < 0.05, ** *p* < 0.01, *** *p* < 0.001).

## 3 RESULTS

### 3.1 CPPs isolated from E9.5 mouse embryos express markers of CPPs

**FIGURE 1:**
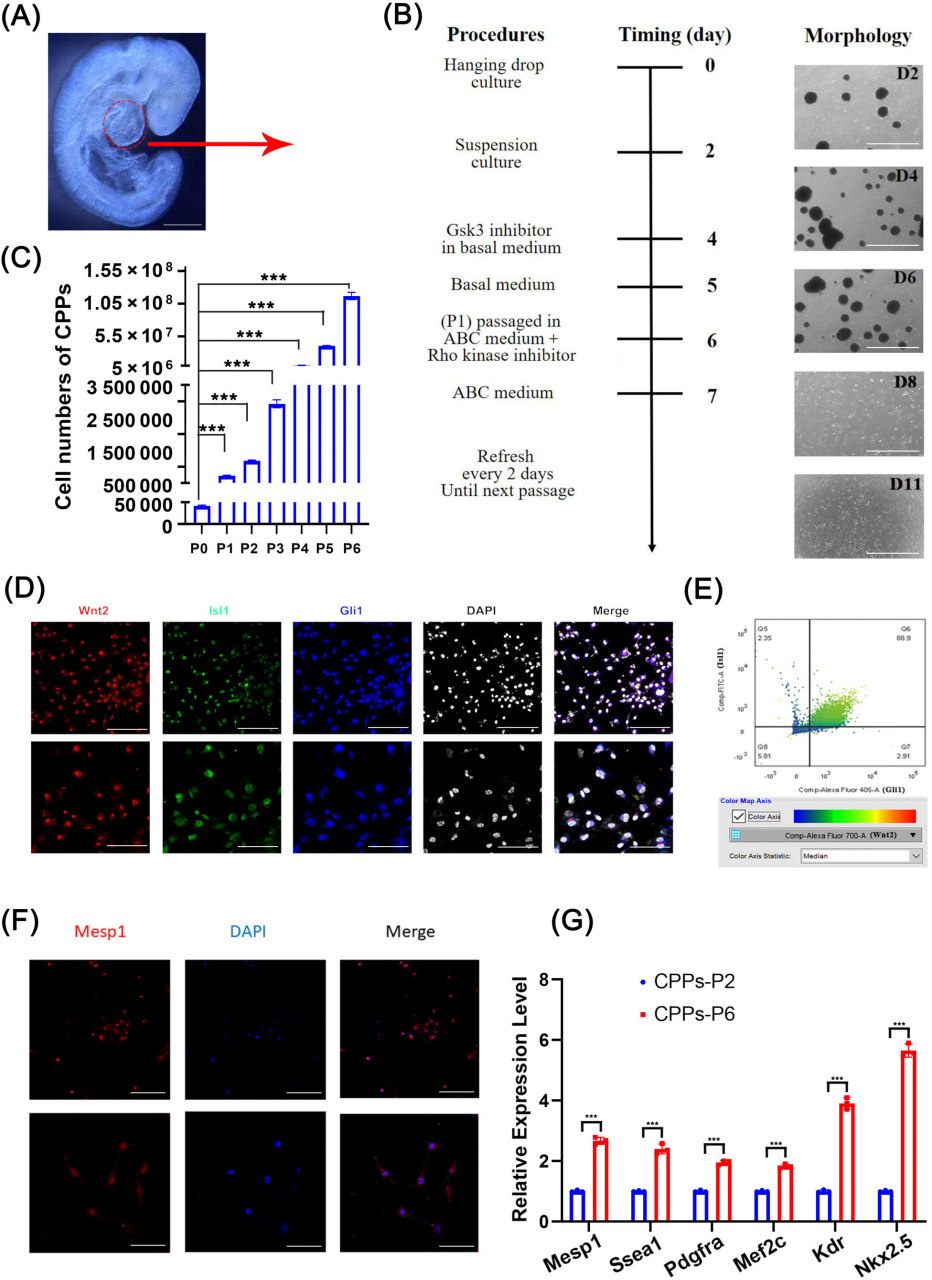
Isolation and identification of cardiopulmonary progenitors (CPPs). (A) Regions for isolating CPPs from 9.5‐day embryos. Scale bar, 2 mm. (B) Schematic diagram of the workflow for CPPs culture in vitro with representative photos. Scale bar, 1 mm. (C) Bar plots showing the cell numbers of CPPs cultured from passage number 0 (P0) to 6 (P6) in vitro. Data are shown as mean ± SEM; *n* = 3 biological replicates per group; ***p < 0.001 (*t*‐test). (D) Identification of CPPs markers (Isl1, Wnt2, and Gli1) by immunofluorescence. Upper images: scale bar, 100 μm; lower images: scale bar, 50 μm. (E) Flow cytometric analysis of CPPs for expression of the CPPs markers Isl1, Wnt2, and Gli1. (F) Immunofluorescence staining of CPPs for the expression of the stemness marker Mesp1. Upper images: scale bar, 200 μm; lower images: scale bar, 100 μm. (G) Identification of mRNA levels of stemness markers, such as *Mesp1*, *Ssea1*, *Pdgfra*, *Mef2c*, *Kdr*, and *Nkx2*.*5* in CPPs. Data are shown as the mean ± SEM; *n* = 3 biological replicates per group; ***p < 0.001 (*t*‐test).

We performed immunofluorescent staining on frozen sections from E9.5 fetal mice, and observed that the heart region expressed Isl1, Wnt2, and Gli1 (Figure S1A), all of which are markers of CPPs. Then, we microdissected the heart region (CPPs region) as illustrated in Figure 1A from E9.5 mice, and expanded CPPs through the workflow exhibited in Figure 1B. To maintain the stemness during expansion, we explored the factors added to the medium (Figure S2A–C), and determined the formulation of the ABC medium, which was DMEM/F12 supplemented with 2% B‐27 without vitamin A, 2 mM L‐glutamine, 1% nonessential amino acids, 0.1 mM beta‐mercaptoethanol, 2.5% human platelet lysate, 1 μM A83‐01, 50 ng/mL bFGF, and 12 μM CHIR‐99021. The amplification trend shown in Figure 1C indicated that we could obtain 100 million CPPs after six passages in vitro from cells in the CPPs region of a single embryo. The expression of CPPs markers (Isl1, Wnt2, and Gli1) was confirmed by both immunofluorescent staining (Figure 1D) and flow cytometry (Figure 1E), which demonstrated that the purity of CPPs in P6 (passage six) was as high as 88.9%. We also detected the mesoderm marker Mesp1 in P6 cells by immunofluorescence staining (Figure 1F). Moreover, we used RT‐qPCR to detect the mRNA levels of stemness genes, such as *Mesp1*, *Ssea1*, *Pdgfra*, *Mef2c*, *Kdr*, and *Nkx2*.*5*, all of which showed significant increases in P6 compared with P2 (Figure 1G). Meanwhile, the CPPs markers, *Isl1*, *Wnt2*, and *Gli1*, also showed significant increases (Figure S1B).

### 3.2 CPPs isolated from E9.5 have the potential to differentiate into two lineage cells

**FIGURE 2:**
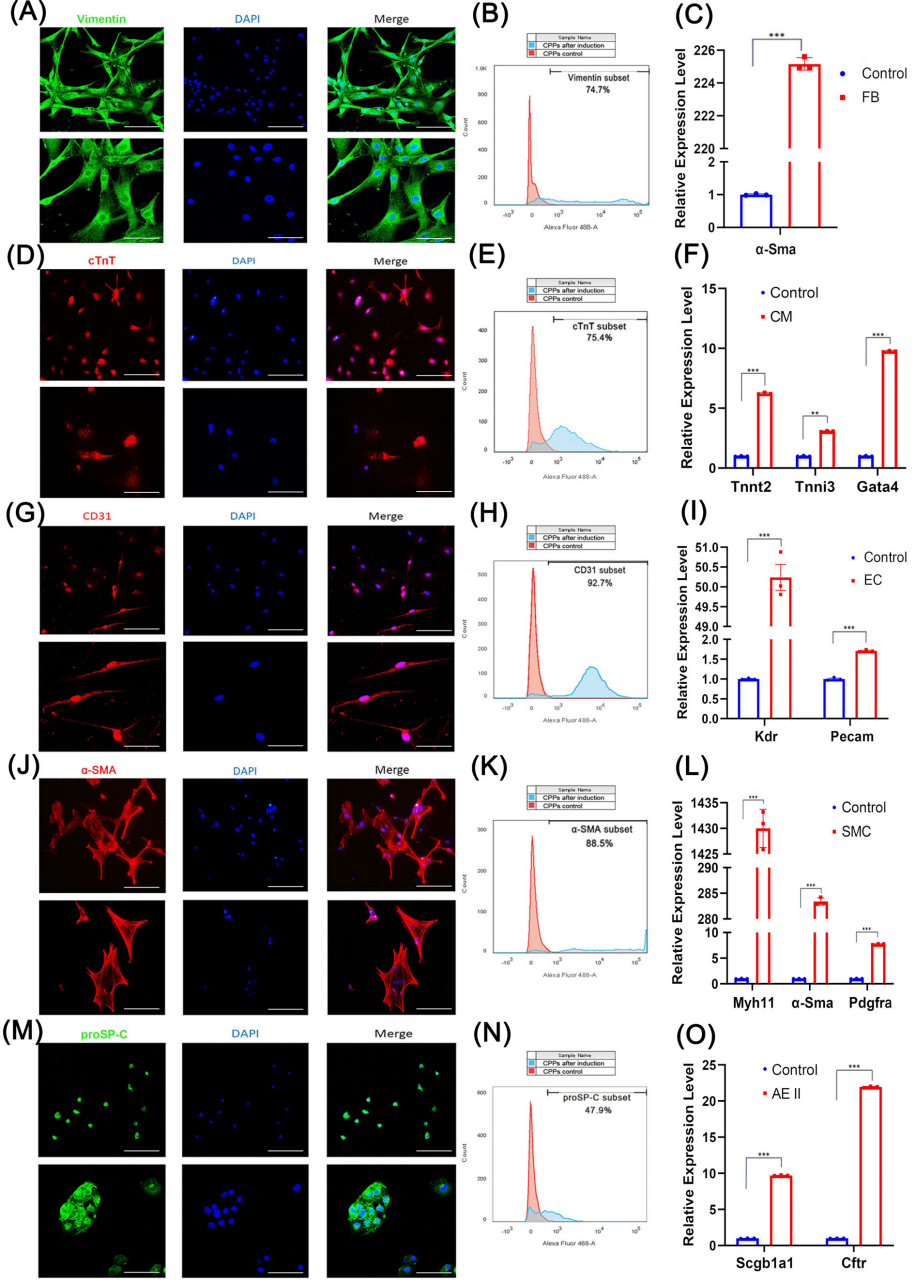
Identification of cardiopulmonary progenitors (CPPs') potential for multiple differentiation. (A–C) Immunofluorescence, flow cytometry and quantitative reverse transcription‐polymerase chain reaction (RT‐qPCR identification of markers for fibroblasts (Vimentin for immunofluorescence and flow cytometry, and *α‐SMA* for RT‐qPCR) in CPPs inducted into fibroblasts. (D–F) Immunofluorescence, flow cytometry and RT‐qPCR identification of markers for cardiomyocytes (cTnt for immunofluorescence and flow cytometry, and *Tnnt2*, *Tnnl3*, and *Gata4* for RT‐qPCR) in CPPs induced into cardiomyocytes. (G–I) Immunofluorescence, flow cytometry and RT‐qPCR identification of markers for endothelial cells (CD31 for immunofluorescence and flow cytometry, and *Kdr* and *Pecam* for RT‐qPCR) in CPPs induced into endothelial cells. (J–L) Immunofluorescence, flow cytometry and RT‐qPCR identification of markers for smooth muscle cells (α‐SMA for immunofluorescence and flow cytometry, and *Myh11*, *α‐Sma*, and *Pdgfra* for RT‐qPCR) in CPPs induced to smooth muscle cells. (M–O) Immunofluorescence, flow cytometry and RT‐qPCR identification of markers for type II alveolar epithelial cells (proSP‐C for immunofluorescence and flow cytometry, and *Scgb1a1* and *Cftr* for RT‐qPCR) in CPPs induced to type II alveolar epithelial cells. For (A, D, G, J, and M), upper images: scale bar, 200 μm; lower images: scale bar, 100 μm. For (G, F, I, L, and O), data are shown as the mean ± SEM.; *n* = 3 biological replicates per group; **p < 0.01, ***p < 0.001 (*t*‐test). CM, cardiomyocyte; EC, endothelial cells; FB, fibroblasts; SMC, smooth muscular cell.

To assess the potential of CPPs for differentiation, we arranged cell assays to differentiate CPPs into five cell types (fibroblasts [FB], CMs, EC, smooth muscle cells, and type II alveolar epithelial cells) according to different methods, as illustrated in Figure S3. Differentiated cells were collected on Day 6 or 12 after induction, and their markers were confirmed by flow cytometry, immunofluorescence staining, and RT‐qPCR. Images from immunofluorescence staining presented positive cells expressing the corresponding markers of FB (Figure 2A, Vimentin), CMs (Figure 2D, cTnT), EC (Figure 2G, CD31), smooth muscle cells (Figure 2J, alpha‐smooth muscle actin [α‐SMA]) and alveolar epithelial cells (Figure 2M, prosurfactant protein C [proSP‐C]), all of which showed that CPPs differentiated into FB, CMs, EC, smooth muscle cells, and type II alveolar epithelial cells successfully. In line with the observations, data from RT‐qPCR not only suggested the potent of CPPs differentiating into the five cell lineages, but also showed us the proportions of successfully differentiated cells were 74.7% (Figure 2B), 75.4% (Figure 2E), 92.7% (Figure 2H), 88.5% (Figure 2K), and 47.9% (Figure 2N), as indicated by flow cytometry. Moreover, the mRNA levels of specific markers for each cell type were up‐regulated remarkably after induction (Figure 2C,F,I,L,O). For example, in comparison with controls, the expression level of CMs marker, *Tnnt2*, increased 6.18‐fold (Figure 2F); the marker of EC, *Kdr*, increased 49.81‐fold (Figure 2I); and the marker of type II alveolar epithelial cells, *Scgb1a1*, increased 9.65‐fold (Figure 2O). Taken together, these data indicated that CPPs exerted their potential to differentiate into CMs, FB, EC, smooth muscle cells, and alveolar epithelial cells in vitro.

### 3.3 CPPs are hybrids exhibiting fibrotic and endothelial states

**FIGURE 3:**
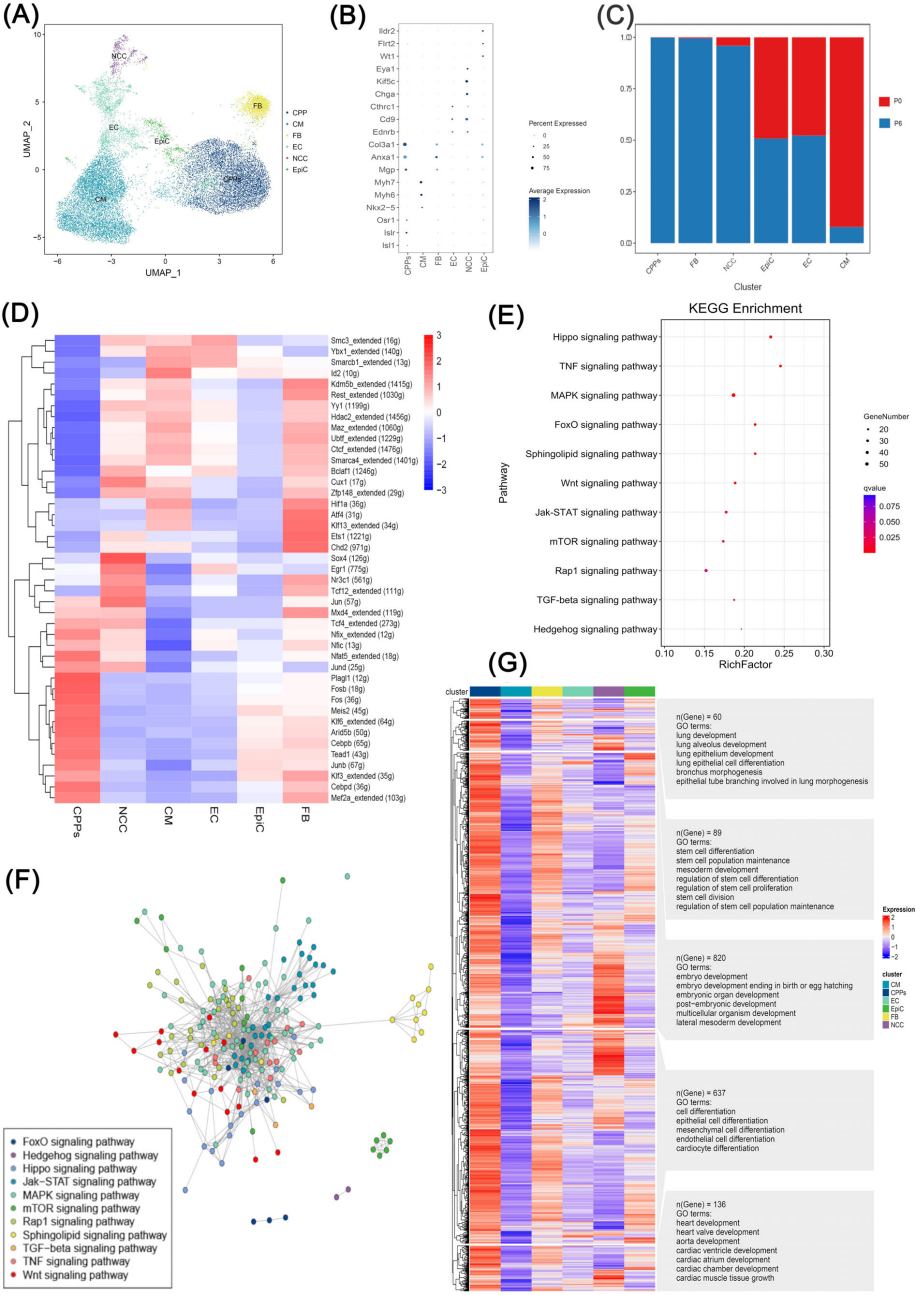
Molecular characteristics and heterogeneity of cardiopulmonary progenitors (CPPs). (A) UMAP plots showing the clusters of CPPs, cardiomyocytes (CM), fibroblasts (FB), endothelial cells (EC), neural crest cells (NCC), and epicardial cells (EpiC). Dots: single cells. (B) Bubble chart of the marker genes for CPPs, CM, FB, EC, NCC, and EpiC in the cell clusters. The dot size and colour intensity represent the gene expression percentage and the average expression levels of the cells within a cluster, respectively. A *p* < 0.05 for all displayed results. (C) Bar plots showing the proportions of each cell type in each sample. (D) Heatmap showing the activated transcription factors (TFs) predicted by SCENIC. For each cell type, the top 10 −log (*p*‐value) specific TFs activated are shown, which are ranked by the number of cells. Columns are individual cells and rows are individual genes. Red: activated; blue or white: not activated. A *p* < 0.05 for all displayed results. (E) KEGG analysis of the differentially expressed genes in CPPs. *p* < 0.05 for all displayed results. (F) Interactive network diagrams showing the specific cell signalling pathways in which the differentially up‐regulated genes were located in CPPs. A *p* < 0.05 for all displayed results. (G) GO analysis of the differentially up‐regulated genes in CPPs. *p* < 0.05 for all displayed results.

**FIGURE 4:**
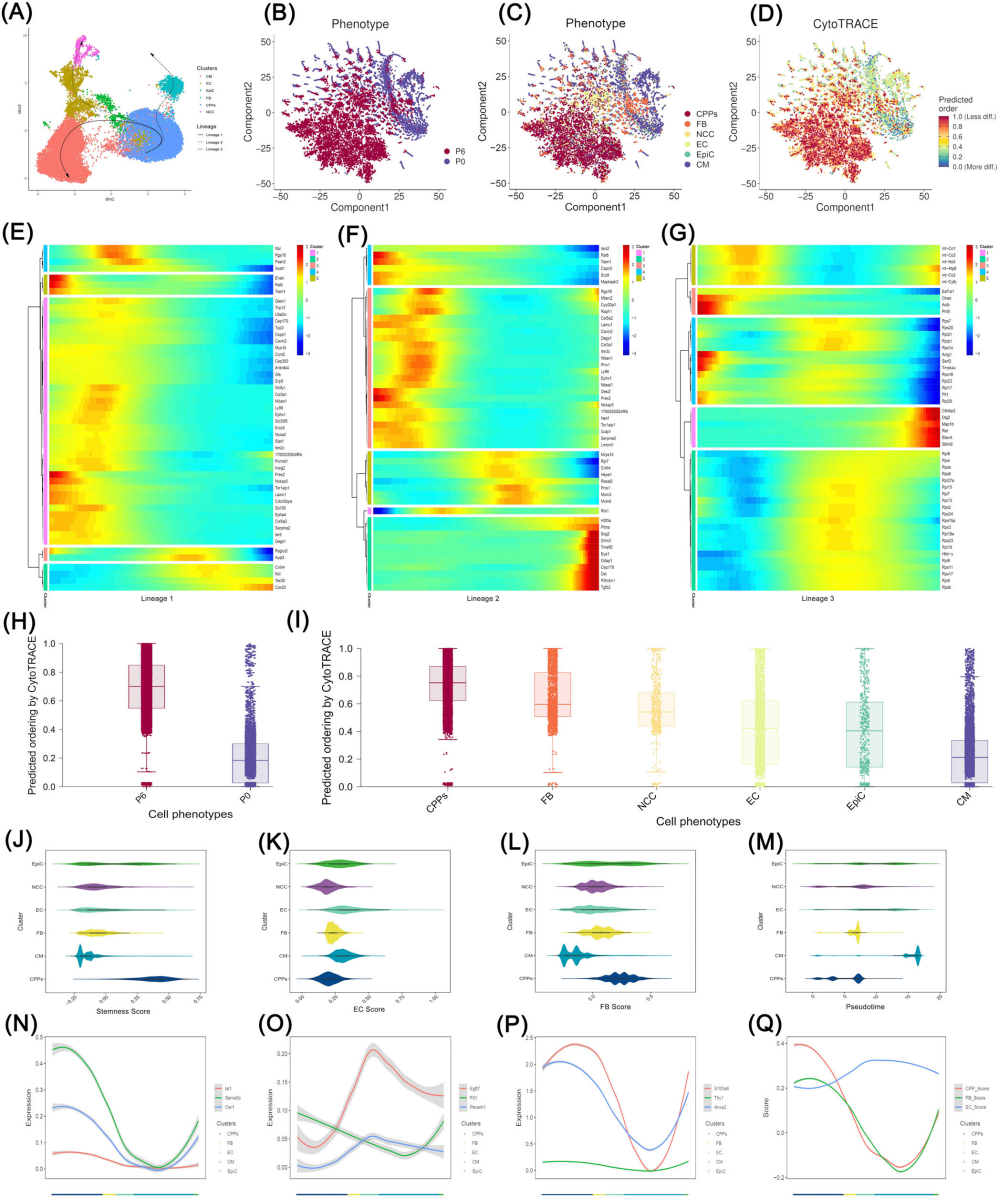
Differentiation trajectories and hybrid fibrotic, epithelial and endothelial states of cardiopulmonary progenitors (CPPs). (A) Pseudotime analysis of CPPs through Slingshot as shown in the UMAP plot. The Slingshot result with the lines indicating the trajectories of lineages and the arrows indicating manually added directions of the pseudotime. Dots: single cells; colours: cell types. (B–D) Pseudotime analyses of CPPs by CytoTRACE. (B) shows the location of P0 and P6, while (C) shows the location of different cell clusters. Dots: single cells; colours: cell types. (D) demonstrates the different levels of differentiation in all cells. Dots: single cells; Colours: loess‐smoothed expression (red, less differentiated; blue, more differentiated). (E–G), Heatmaps showing the relative expression of genes displaying significant changes along the pseudotime axis of each lineage [Lineages 1–3 corresponding to (E–G), respectively]. Colours: loess‐smoothed expression (red, high; blue, low). The columns represent the cells of each lineage. Rows represent genes ordered by their peak expression along the pseudotime axis. *p* < 0.05 for all displayed results. (H,I), Boxplots showing CytoTRACE values for P0 and P6 (H), or for CPPs, CM, FB, EC, NCC, and EpiC (I). All plots are identically ordered by CytoTRACE. *p* < 0.05 for all displayed results. (J–M), The values of the Stemness score (J), the EC score (K), and the FB score (L), as well as the cell proportion levels of each cell cluster along the pseudotime axis (M). (N–Q), Line graphs showing the expression levels of representative TFs of each cell cluster along the axis displaying different cell types. A *p* < 0.05 for all displayed results. CM, cardiomyocyte; EC, endothelial cells; EpiC, epicardial cells; FB, fibroblasts; NCC, neural crest cells.

To decode CPPs population by single‐cell level, we employed scRNA‐seq and identified six cell clusters named CPPs, CMs, FB, EC, neural crest cells, and epicardial cells (EpiC) in P0 and P6 samples (Figure 3A) using representative markers for each cell type (Figure 3B). For example, we used *Isl1*, *Islr*,^32^ *Osr1*,^33^ and other genes for the identification of CPPs. Among the six‐cell clusters, CPPs showed the biggest increase from P0 to P6, as shown in Figure 3C. So, the majority of cells in P6 were CPPs. To further analyse the difference in activated TFs between these cell clusters, we used SCENIC and found several TFs, including *Mef2a*, *Junb*, *Meis2*, *Klf6*, and *Fos* (Figure 3D). The DEGs in CPPs were located in cell signalling pathways related to cell proliferation and differentiation, such as the Hippo signalling pathway, the MAPK signalling pathway, the Wnt signalling pathway, and the Jak–STAT signalling pathway, as indicated by KEGG enrichment (Figure 3E). The interactive network diagrams demonstrated that the genes located in the MAPK signalling pathway and the Jak–STAT signalling pathway were in the core of these interaction networks (Figure 3F). Moreover, GO analysis showed that the functions of up‐regulated genes specific for CPPs belonged to the categories of stem cell maintenance and differentiation, development of embryos, hearts and lung, and differentiation of cells (e.g., CMs, EC, epithelial cells, and mesenchymal cells; Figure 3G). Taken together, these results implied that CPPs have the potential of stem cells to differentiate into other cell types. Next, we reconstructed the developmental progression of these six‐cell clusters by Slingshot and CytoTRACE.^34^ Figure 4A shows that cells in the lower right corner, which were CPPs, represented the starting point of the trajectories, and Slingshot data identified three lineage trajectories among the six‐cell populations during pseudotime developmental progression. Lineages 1, 2, and 3 demonstrated the three routes that CPPs differentiated into CM, EC, and FB, respectively (Figure 4A). Consistently, pseudotime analyses by CytoTRACE indicated that CPPs were in a less differentiated state than other cell types (Figure 4B–F,I), and P6 was in a less differentiated state than P0 (Figure 4H). Cells could be divided into five stages in each lineage trajectories shown in Figure 4A, according to the differences in the peak expressing genes along the pseudotime (Figure 4E–G). For example, during differentiation into CM, cells expressing *Enah*, an actin regulator,^35^ or *Ralb*, which support mitotic progression^36^ decreased their expression levels gradually, while in contrast, another group of cells expressed increasing *Cox20*, which is related to the assembly of mitochondrial complex IV^37^ (Figure 4E). Inspired by Shu Zhang's paper,^38^ we calculated the values of the stemness score, the EC score, and the FB score (Figure 4J–L), as well as the proportion of cells in each cell cluster along the pseudotime axis (Figure 4M). Meanwhile, the expression levels of representative genes used for the stemness score, the EC score and the FB score were shown in Figure 4N–P along the axis displaying different cell types, respectively. These diagrams indicated that CPPs expressed stemness markers along with some of the EC and FB markers (Figure 4N–P), implying that CPPs were a group of cells presenting hybrid state of stem cells, EC and FB. In concordance with this finding, the score of CPPs for stemness was the highest, while CPPs also presented a certain EC and FB score (Figure 4J–L,Q). All of these results suggested that CPPs existed in a state expressing not only the stemness markers but also some of the fibrotic and endothelial markers, fitting the term of a hybrid F/E state, which was similar to a population of stem cells found in another study.^38^

### 3.4 CPPs improve heart function after MI via CPPs‐Exo

After extracting the exosomes in the supernatant of CPPs culture medium by ultracentrifugation (Figure S4A), we confirmed the average diameter of the particles by NTA, which was 136.9 ± 5.9 nm in diameter, with particle concentrations of ~4.37 × 10^9^/mL (Figure S4B). The typical cup‐shaped appearance of microvesicles in CPPs‐Exo was confirmed by employing TEM (Figure S4C). The surface markers of exosomes, CD63, Alix, and Hsp70 were confirmed in CPPs‐Exo by Western blot (Figure S4D). With the help of miRNA‐seq, we ranked the expression levels and obtained the top 30 miRNAs that were highly expressed (Top 30 miRNAs) in CPPs‐Exo (Figure S4E). GO enrichment implied that CPPs‐Exo mostly regulated genes related to protein binding (Figure S4F). KEGG enrichment indicated that CPPs‐Exo are potentially involved in cell proliferation and differentiation, such as the Wnt signalling pathway, PI3K‐Akt signalling pathway, and MAPK signalling pathway (Figure S4G). These data suggest that exosomes derived from CPPs (CPPs‐Exo) exhibit regulatory effects on cell signalling pathways related to cell proliferation and differentiation.

**FIGURE 5:**
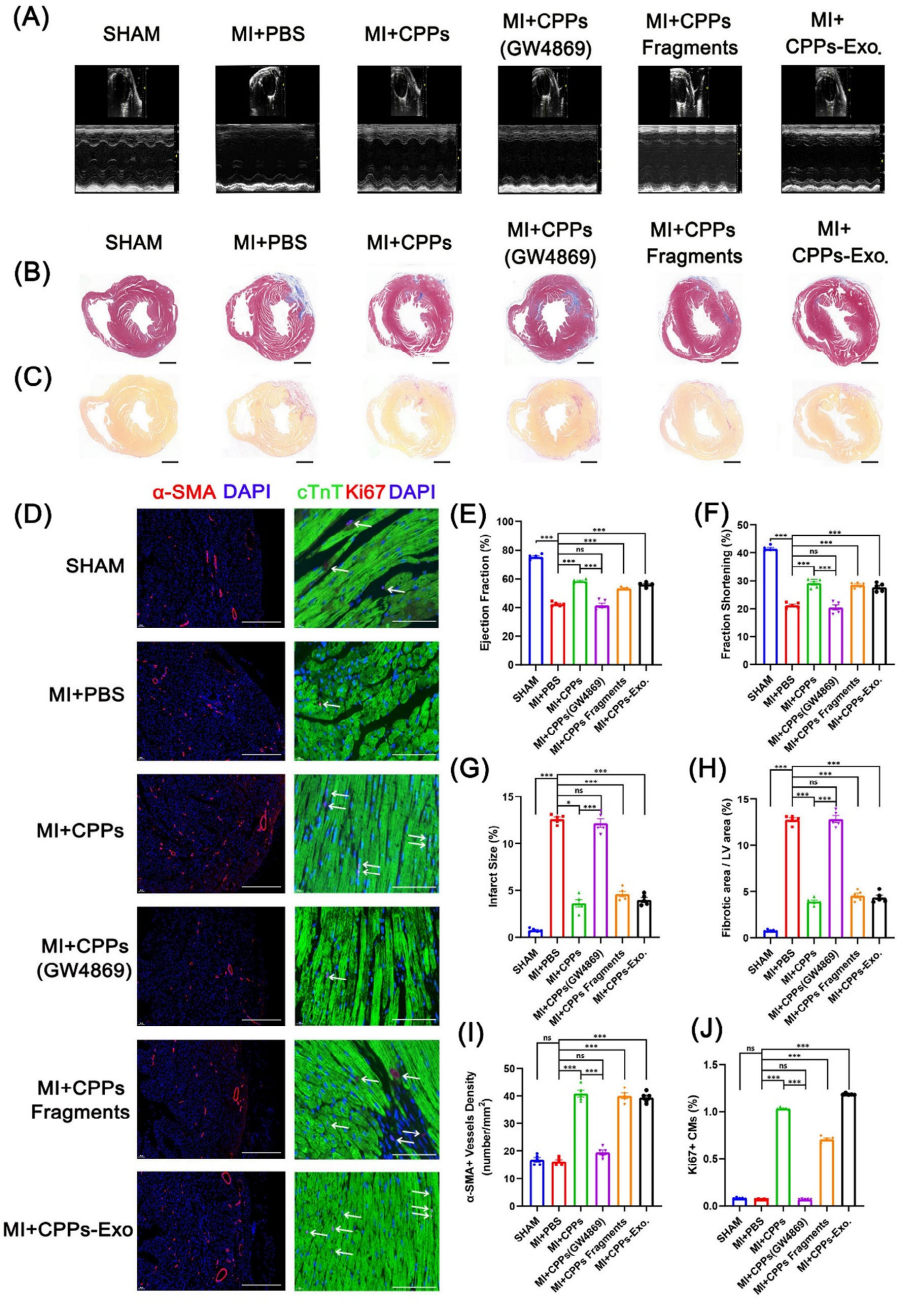
Restoration of heart function after myocardial infarction (MI) by cardiopulmonary progenitor cells (CPPs) through CPPs exosomes (CPPs‐Exo). (A) Representative photographs of M‐mode echocardiography. Quantitative analysis of echocardiography is shown in (E,F). (B) Representative Masson's trichrome‐stained sections of murine hearts from six groups (SHAM, MI + PBS, MI + CPPs, MI + CPPs treated with GW4869, MI + CPPs fragments and MI + CPPs‐Exo, *n* = 5 per group). Scale bar, 1 mm. Quantitative analyses of the infarct size are shown in (G). (C) Representative images of Sirius red staining at 42 days after ligation of the hearts in 6 groups (SHAM, MI + PBS, MI + CPPs, MI + CPPs treated with GW4869, MI + CPPs fragments and MI + CPPs‐Exo, *n* = 5 per group). Scale bar, 1 mm. The percentage of fibrotic size is shown in (H). (D) Immunofluorescence detection of α‐SMA in sections of murine hearts from 6 groups (SHAM, MI + PBS, MI + CPPs, MI + CPPs treated by GW4869, MI + CPPs fragments and MI + CPPs‐Exo, *n* = 5 per group). Scale bar, 500 μm. Immunofluorescence detection of Ki67 and cTnT in heart sections of 6 groups (SHAM, MI + PBS, MI + CPPs, MI + CPPs treated with GW4869, MI + CPPs fragments and MI + CPPs‐Exo, *n* = 5 per group). Scale bar, 250 μm. (I) Determination of vessel density by quantifying the number of structures that expressed α‐SMA in an area of 1 mm^2^. (J) Determination of the Ki67+/cTnT+ cardiomyocyte ratio by quantifying the number of Ki67+/cTnT+ cells and cTnT+ cells. For (E–J), data are shown as the mean ± SEM; *n* = 5 biological replicates per group; *p < 0.05, ***p < 0.001, ns: not significant (*t*‐test).

There was no formation of teratomas in nude mice 3 months later after subcutaneous injection of CPPs (Figure S5A,B), implying that we could inject CPPs into the hearts of MI mice without the risk of teratoma formation. Moreover, karyotyping analysis was employed to verify the integrity of chromosomes in CPPs (Figure S5C). To trace the injected CPPs, we isolated CPPs from male embryos that contained Y chromosomes, and then chose female mice for the establishment of the MI model. The results from immunofluorescence staining plus Y chromosome probe hybridization showed no Y chromosome in the heart region of MI mice (Figure S6A–E). To assess whether the delivery of CPPs at the time of ischemia induction has functional benefits, we established an MI mouse model. The mice were divided into six groups: the MI + PBS group with permanent ligation of the LAD and injection of PBS in the ischemic region; the sham group, which underwent all surgical procedures of the MI + PBS group except coronary artery occlusion and injection of PBS; MI + CPPs and MI + CPPs (GW4869) groups, in which CPPs (without and with previous incubation with GW4869) were injected directly into the ischemic region of the myocardium, and the MI + CPPs fragments and MI + CPPs‐Exo groups, in which fragments of CPPs and CPPs‐Exo were intramyocardially injected into the ischemic region. Cardiac function was evaluated 6 weeks after MI surgery by cardiac ultrasound (Figure 5A), and the data showed that the left ventricular EF and FS in mice from the MI + CPPs, MI + CPPs fragments and MI + CPPs‐Exo groups were significantly improved, compared with those in mice from the MI + PBS group (Figure 5E,F), indicating that CPPs and their exosomes were beneficial to the infarcted heart. The measurements of EF and FS were nearly equal between the MI + PBS group and the MI + CPPs (GW4869) group, but were markedly lower than those in the SHAM group, implying that the inhibition of exosome biogenesis and release impaired the beneficial effect of CPPs on cardiac function. All of the mice were euthanized 6 weeks after MI or sham surgery, and the heart sections were stained with Masson's trichrome and Sirius Red (Figure 5B,C). The results showed that the two groups, the MI + PBS group and MI + CPPs (GW4869) group, displayed significant differences in infarct size (12.37% and 12.12%, respectively) and fibrotic size (12.56% and 12.77%, respectively) compared with the sham group (Figure 5G,H), implying that cardiac function was worsened in these two groups. However, those measurements in the MI + CPPs, MI + CPPs fragments, and MI + CPPs‐Exo groups were all below 5%, markedly lower than those in the MI + PBS group (Figure 5G,H), demonstrating the beneficial effect of CPPs and CPPs‐Exo on the attenuation of scar formation. Taken together, these results suggested that CPPs, CPPs fragments, and CPPs‐Exo were associated with similar improvements in cardiac function, and decreased infarct size and fibrotic size when delivered into the infarcted region of murine hearts. Moreover, inhibition of exosome synthesis and release hindered the improvements directed by CPPs.

To assess the effects of CPPs on angiogenesis and CM proliferation, we performed immunofluorescence staining for the marker of vascular smooth muscle cells α‐SMA and the marker of proliferative cells Ki67 in heart tissue slices (Figure 5D). The α‐SMA+ vessel density increased to nearly 40 per mm^2^ in the MI + CPPs, MI + CPPs fragments and MI + CPPs‐Exo groups but was below 20 per mm^2^ in the SHAM, MI + PBS and MI + CPPs (GW4869) groups (Figure 5I). The proliferative CMs, presented as cells co‐stained by cTnT and Ki67 antibodies (Figure 5D), exhibited significantly higher proportion in the MI + CPPs, MI + CPPs fragments and MI + CPPs‐Exo groups (1.03%, 0.71%, and 1.18%, respectively), while only ~0.06% in the SHAM, MI + PBS, and MI + CPPs (GW4869) groups (Figure 5J). Therefore, it could be concluded that the beneficial effects of CPPs on cardiac function and scar formation were due to the promotion of CM proliferation and angiogenesis.

### 3.5 CPPs‐Exo promotes angiogenesis and cardiomyocytic proliferation

**FIGURE 6:**
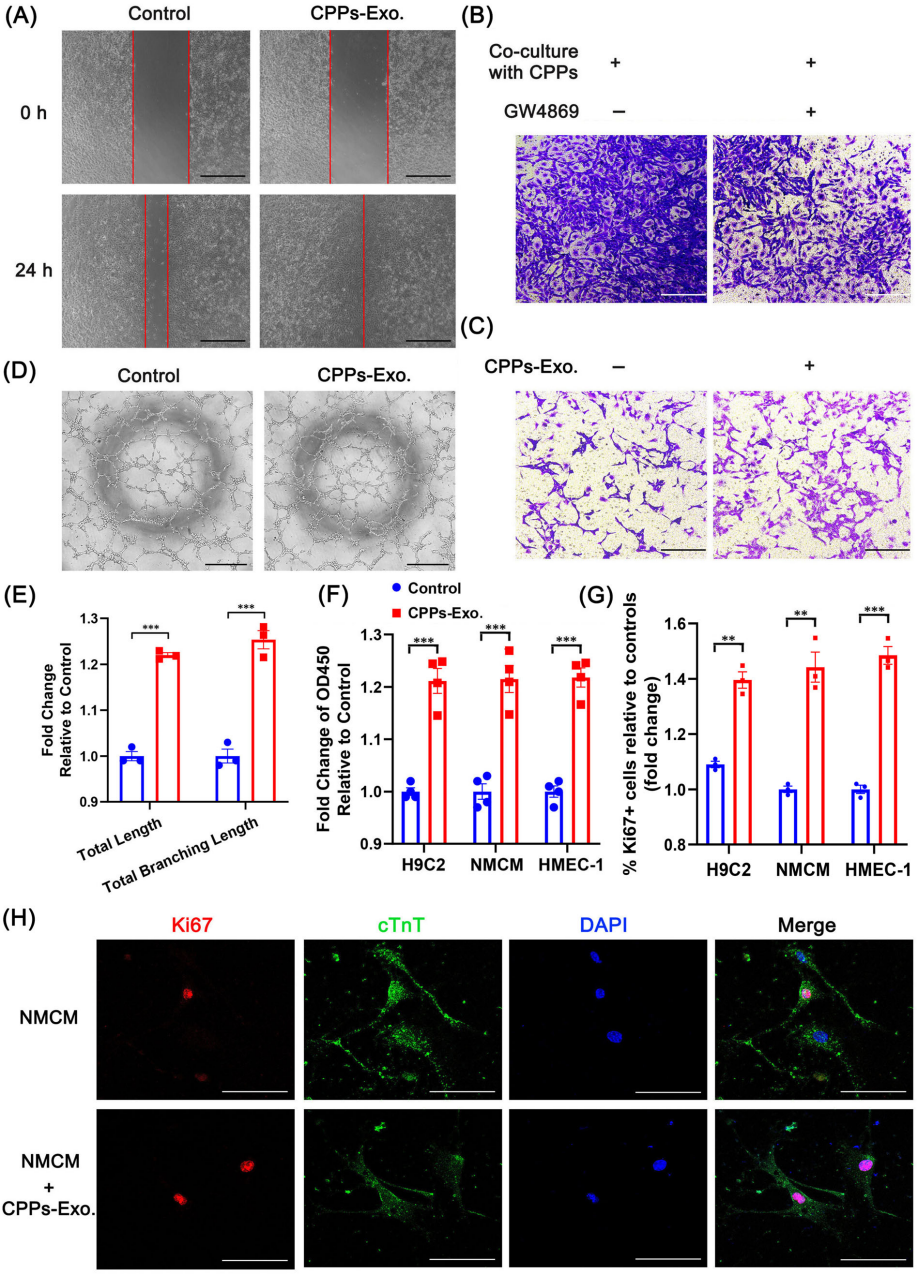
Promotion of cardiomyocyte proliferation and endothelial function by cardiopulmonary progenitor cells (CPPs) through CPPs exosomes (CPPs‐Exo). (A) Representative images showing the healing of cell scratches at 0 and 24 h after addition of CPPs‐Exo to endothelial cells. Scale bar, 500 μm. (B) Representative images of invaded cells at the bottom of the Transwell chamber when endothelial cells were co‐cultured with CPPs with or without GW4869. Scale bar, 250 μm. (C) Representative images of invaded cells at the bottom of the Transwell chamber when endothelial cells were treated with or without CPPs‐Exo. Scale bar, 250 μm. (D) Representative images of tube formation by endothelial cells with or without the addition of CPPs‐Exo. Scale bar, 500 μm. (E) Analysis of the tube formation by Image J. Data are shown as the mean ± SEM; *n* = 3 biological replicates per group; ***p < 0.001 (*t*‐test). (F) Change in the proliferation of cardiomyocytes (H9C2 and NMCM) and endothelial cells after CPPs‐Exo treatment, as determined by CCK‐8 assay. Data are shown as the mean ± SEM; *n* = 4 biological replicates per group; ***p < 0.001 (*t*‐test).(G) The histogram showed fold changes in percentage of Ki67 positive cells in cardiomyocytes (H9C2 and NMCM) and endothelial cells by flow cytometry. Data are shown as the mean ± SEM; *n* = 3 biological replicates per group; **p < 0.01, ***p < 0.001 (*t*‐test). (H) Representative images of the expression of Ki67 and cTnT in NMCM incubated with or without CPPs‐Exo by immunofluorescence staining. Scale bar, 100 μm.

To explore the effects of CPPs‐Exo on EC functions, we conducted experiments on proliferation, migration, invasion, and tube formation. As illustrated in Figure 6A, adding CPPs‐Exo to EC HMEC‐1 (100 μg/mL), significantly increased the healing of the scratch, which was nearly closed compared with control group, after 24 h of scratch. Employing Transwell assays, we found that there was a sharp decrease in the number of cells that passed through the Transwell chamber when HMEC‐1 cells were co‐cultured with CPPs after the addition of 20 μM GW4869, an inhibitor of exosome biogenesis and release, compared with controls (Figure 6B), indicating that CPPs may exert their invasion‐promoting effect by exosomes. Furthermore, we confirmed that the number of invaded cells increased after adding CPPs‐Exo to HMEC‐1 cells compared with controls (Figure 6C), proving that CPPs‐Exo possesses the ability to promote EC invasion. Representative images of tube formation were exhibited in Figure 6D, and the data analysed by Image J demonstrated that the total length and total branching length in the CPPs‐Exo group were significantly increased compared with those in the control group, both showing a nearly 1.2‐fold increase (Figure 6E). By employing CCK‐8 experiments, we found that the proliferative HMEC‐1 cells increased significantly (1.22‐fold increase) after the addition of 100 μg/mL CPPs‐Exo, as compared with that in the controls (Figure 6F). In line with this result, flow cytometry detecting Ki67‐positive cells in HMEC‐1 cells treated with CPPs‐Exo demonstrated similar increase (Figure 6G). Conclusively, CPPs‐Exo enhances the function of EC by promoting proliferation, migration, invasion, and tube formation. For CMs, CPPs‐Exo significantly promoted the proliferation of H9C2 and NMCM, compared with controls (Figure 6F 1.21‐ and 1.24‐fold increases, respectively), and the percentages of Ki67‐positive cells increased significantly compared with controls (Figure 6G). Likewise, images from immunofluorescent staining confirmed that Ki67+ (red) cells in cTnT+ NMCM cells were increased after adding CPPs‐Exo (Figure 6G).

### 3.6 The effects on pro‐CM‐proliferation and pro‐angiogenesis through miR‐27b‐3p derived from CPPs‐Exo

**FIGURE 7:**
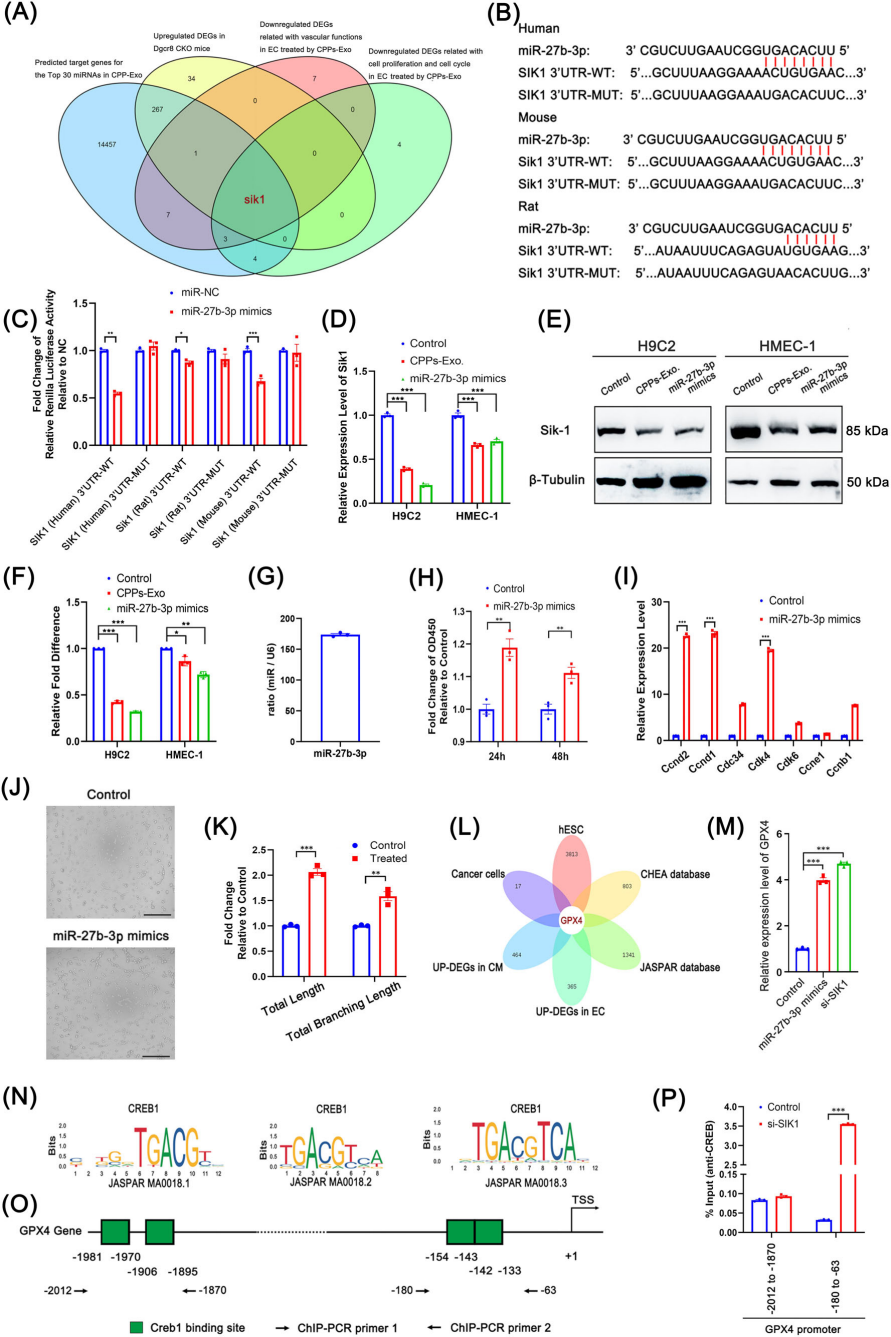
The effects on pro‐cardiomyocyte‐proliferation and pro‐angiogenesis through miR‐27b‐3p derived from cardiopulmonary progenitor cells (CPPs) through CPPs exosomes (CPPs‐Exo). (A) Venn diagram showing the overlapping gene (*Sik1*) between four gene sets, that are predicted target genes for Top 30 miRNAs in CPP‐Exo, down‐regulated differentially expressed genes (DEGs) related to vascular functions or cell proliferation and the cell cycle in EC treated with CPPs‐Exo, and up‐regulated DEGs in *Dgcr8* CKO mice. (B) Diagram showing the binding sites of miR‐27b‐3p to the 3′UTR regions of *Sik1* genes from human, mice and rats and the corresponding mutant binding sites. (C) The histogram demonstrating the results of the dual‐luciferase reporter assay in 293 T cells transfected with miR‐27b‐3p mimics along with plasmids endowing the wild type or mutant type of the miR‐27b‐3p binding sites. Data are shown as the mean ± SEM; *n* = 3 biological replicates per group; *p < 0.05, **p < 0.01, ***p < 0.001 (*t*‐test). (D) Histogram exhibiting the mRNA levels of *Sik1* in H9C2 or HMEC‐1 cells transfected with miR‐27b‐3p mimics or cultured with CPPs‐Exo. Data are shown as the mean ± SEM; *n* = 3 biological replicates per group; ***p < 0.001 (*t*‐test). (E) The bands in this diagram showing the protein levels of *Sik1* in H9C2 or HMEC‐1 cells transfected by miR‐27b‐3p mimics or cultured with CPPs‐Exo. (F) Quantification of e via densitometry. Densitometry measurements were normalized to β‐tubulin levels as a control for unequal loading, and all quantified results were normalized to measurements in the control. (G) Histogram demonstrating the result of miR‐27b‐3p expression level relative to that of U6 in CPPs‐Exo. (H) Histogram demonstrating the results of the CCK8 assay in H9C2 cells transfected with or without miR‐27b‐3p mimics. Data are shown as the mean ± SEM; *n* = 3 biological replicates per group; **p < 0.01 (*t*‐test). (I) Histogram exhibiting the mRNA levels of genes (*Ccnd2*, *Ccnd1*, *Cdc34*, *Cdk4*, *Cdk6*, *Ccne1*, and *Ccnb1*) related to the cell cycle in H9C2 cells transfected with miR‐27b‐3p mimics. Data are shown as the mean ± SEM; *n* = 3 biological replicates per group; ***p < 0.001 (*t*‐test). (J) Pictures representing the results of the tube formation assay in HMEC‐1 cells transfected with miR‐27b‐3p mimics. Scale bar, 500 μm. (K) The histogram exhibiting the analytic results of i on total length and total branching length by the software named Image J. Data are shown as the mean ± SEM; *n* = 3 biological replicates per group; **p < 0.01, ***p < 0.001 (*t*‐test). (L) Venn diagram showing the overlapping gene (*Gpx4*) between up‐regulated DEGs in cardiomyocyte (CM) or endothelial cells (EC) transfected with miR‐27b‐3p mimics, predicted binding genes of CREB1 in cancer cells or human embryonic stem cell, or from the CHEA and JASPAR databases. (M) Histogram exhibiting the mRNA levels of *Gpx4* in HMEC‐1 cells transfected with miR‐27b‐3p mimics or si‐*Sik1*. Data are shown as the mean ± SEM; *n* = 3 biological replicates per group; ***p < 0.001 (*t*‐test). (N,O) Diagram representing the binding sequences of CREB1 (N), and the predicted binding sites in the promoter region of the *Gpx4* gene (O). (P) Histogram showing the chromatin immunoprecipitation (ChIP)‐quantitative polymerase chain reaction results for CREB1 and the predicted binding sites in the promoter region of the *Gpx4* gene. Data are shown as the mean ± SEM; *n* = 3 biological replicates per group; ***p < 0.001 (*t*‐test).

To further reveal the mechanism underlying the functions of CPPs‐Exo in cardiomyocytic proliferation and angiogenesis, we compared the predicted target genes for the TOP 30 miRNAs in CPPs‐Exo, with three other gene lists from up‐regulated DEGs in *Dgcr8* conditional knockout (CKO) mice,^39^ and from down‐regulated DEGs in EC cells treated with CPPs‐Exo related to cell proliferation and cell cycle or vascular function, and identified the overlapped gene *Sik1*, as shown in the Venn diagram (Figure 7A). Then, we identified miR‐27b‐3p, which was the only predicted miRNA that could target *Sik1* mRNA among the TOP 30 miRNAs of CPPs‐Exo. The results of the dual‐luciferase reporter assay (Figure 7C) confirmed that miR‐27b‐3p could bind the predicted binding regions shown in Figure 7B, which included three species: human, mouse, and rat. Moreover, miR‐27b‐3p had the potential to down‐regulate the expression level of *Sik1* in both CM and EC at the mRNA and protein levels, and similar results were demonstrated by adding CPPs‐Exo, as implied by Figure 7D–F. We also examined the expression level of miR‐27b‐3p in CPPs‐Exo, which showed more than 176‐fold change compared with U6 expression (Figure 7G). The result was in line with the sequencing data of miRNAs in CPPs‐Exo, which showed that miR‐27b‐3p expressed highly as one of the top 5 most abundant miRNAs (Figure S4). In CM, miR‐27b‐3p promoted cell proliferation (Figure 7H) by increasing the expression of cell cycle‐related genes, such as *Ccnd2*, *Ccnd1*, *Cdc34*, *Cdk4*, *Cdk6*, *Ccne1*, and *Ccnb1* (Figure 7I). In EC, miR‐27b‐3p promoted tube formation in HMEC‐1 cells transfected with miR‐27b‐3p mimics (Figure 7J,K). Inspired by the paper showing that SIK1 phosphorylates and deactivates CRTC1 (CREB‐regulated transcription coactivator 1),^40^ resulting in a decrease in CREB1 transcriptional activity,^41^ we looked for potential genes downstream of the SIK1‐CREB1 axis by Venn analysis to compare six gene lists (up‐DEGs in CM or EC transfected by miR‐27b‐3p mimics, predicted genes regulated by CREB1 in cancer cells, or hESC, or in the CHEA database or JASPAR database) and finally found the gene *Gpx4* (Figure 7L). The RT‐qPCR results further confirmed that transfection with either miR‐27b‐3p or si‐*Sik1* increased the expression level of *Gpx4* (Figure 7M), implying that *Gpx4* was downstream of the miR‐27b‐3p‐*Sik1* axis. To further confirm the binding of CREB1 to the promoter region of *Gpx4*, we employed ChIP‐qPCR to assess the four binding sites with three binding motifs predicted by JASPAR (Figure 7N,O), and verified that the region from −180 to −63 of the *Gpx4* promoter was the binding site for CREB1 (Figure 7P). Taken together, CPPs‐Exo was endowed with the potential for pro‐CM‐proliferation and pro‐angiogenesis partially through the up‐regulation of *Gpx4* by miR‐27b‐3p via the SIK1‐CREB1 axis.

## 4 DISCUSSION

In this study, we investigated whether CPPs could repair an injured heart after myocardial infarct. First, we isolated CPPs from E9.5 murine embryos, maintained CPPs stemness while expanding them with ABC medium, and confirmed that CPPs harbour the unique potential to differentiate into two lineages of cells, which is in line with the latest publication showing that Isl1+ CPPs give rise to SMC, EC, EpiC, and alveolar myofibroblasts.^19^ Data from scRNA‐seq demonstrated that CPPs we obtained were a population mainly composed of heterogenic stem cells expressing CPPs marker Isl1, and exhibited a hybrid fibroblastic and endothelial state, which is similar to another study that found that pituitary stem cells are in a hybrid epithelial and mesenchymal state.^38^ Additional analysis is needed to demonstrate the membrane markers for our CPPs, and verification experiment should be performed.

There are substantial studies on heart regeneration by cell therapies, even in clinical trials, such as SCIPIO‐NCT00474461, CADUCEUS‐NCT00893360, and CAREMI‐NCT02439398. Among them, the ESCORT trial performed the first transplants of hESC‐derived cardiac progenitor patches in patients with ischemic cardiomyopathy and reported no tumour formation,^42^ which confirmed the safety of these stem cells for translation. In line with this finding, we confirmed that no teratomas formed after subcutaneously injecting CPPs into nude mice, ensuring that CPPs are safe for injection into the heart.

After intramyocardial CPPs administration, the heart function improved significantly, as indicated by our results, such as a decrease in infarct size and fibrotic size, and a dramatic increase in the number of α‐SMA‐positive blood vessels and Ki67‐positive CM, compared with controls. In conclusion, CPPs alleviate heart injury from MI by promoting angiogenesis and myocardial cell proliferation. These findings reveal the previously unrecognized role of CPPs in promoting recovery of cardiac function from progressive worsening when delivered at the early phase of MI.

Many stem cell types, such as skeletal myoblasts, bone marrow‐derived stem cells, embryonic stem cells, induced pluripotent stem cells, and endogenous cardiac stem cells, have been investigated as candidates to improve cardiac function after MI. Despite inconsistent results in animal models, most of the studies showed an overall beneficial effect on cardiac regeneration.^43^ However, it is difficult to compare our approach with existing therapeutic modalities, and some suggestions have been offered. First, the therapeutic potential of stem cells should be proven in two or more independent research centres, as proposed by the CAESAR consortium, using detailed protocols (‘CAESAR protocols’) for measuring infarct size with a level of rigour analogous to multicentre randomized clinical trials.^44^ Second, standardization of these variables (e.g., times of application after MI, doze of stem cells) is highly needed and would allow better comparison between different stem cell types and results obtained from different research groups. Despite of these difficulties, we can still be inspired by other approaches. For example, it is reported that intramyocardial injection of human CDC exosomes has resulted in higher exosome retention and efficacy as compared to intracoronary injection.^45^ So, we employed intramyocardial injection instead of intracoronary injection, in order to achieve better efficacy of CPPs‐Exo on injured heart. Moreover, there are emerging combination therapies for MI, such as cell therapy combined with gene therapy,^46^ or pharmacotherapy,^47^ or protein therapy,^48^ or exosome therapy plus gene therapy,^49^ which provide new directions for the future research on CPPs.

In addition to cell‐based therapy, there are cell‐free strategies, such as EVs and exosomes. Studies have illustrated that EVs derived from various stem cells harbour the ability of cardioprotection when delivered in murine models of MI^50^, ^51^ or HF.^52^, ^53^ Our findings confirm that CPPs exert reparative functions through CPPs‐Exo, as blocking exosome release abrogated the functional recovery achieved by delivery of CPPs into the ischemic area of the murine heart. Moreover, CPPs‐Exo delivery at the early phase of MI recapitulates the beneficial effects of their parent cells in the treatment of MI. Thus, we conclude that CPPs‐Exo is indispensable for the functional benefits of CPPs. Consistently, the latest research displays that intracoronary CDC administration improves cardiac function through CDCex‐derived miRNAs as potential paracrine mediators.^54^ Strikingly, a recent clinical trial (NCT04045405) showed that an antisense oligonucleotide drug, CDR132L (a specific antisense oligonucleotide for miR‐132), has a good cardiac repair effect.^55^ All these results imply that miRNAs in exosomes may play vital roles in the functions presented by exosomes.

As miRNAs in exosomes are one of the paracrine mediators and have the potential to be new targets for therapeutic strategies, we employed miRNA‐seq in CPPs‐Exo and bulk RNA‐seq in CMs and ECs incubated with CPPs‐Exo, and found an intersection (the *Sik1* gene) between the potential target gene list of our Top 30 miRNAs and three other gene lists, which were down‐regulated genes related to vascular functions or cell proliferation and the cell cycle in EC cells treated with CPPs‐Exo, and up‐regulated genes in the hearts of E9.5 embryos from *Dgcr8* CKO mice.^39^ SIK1 is a serine/threonine protein kinase that can phosphorylate and deactivate CRTC1,^40^ resulting in a decrease in CREB1 transcriptional activity.^41^ We found that *Sik1* is down‐regulated by miR‐27b‐3p, and then the transcriptional activity of CREB1 would be improved, in line with observations linking SIK1 to CREB transcriptional activity.^56^

To reveal the downstream genes regulated by CREB1 in our study, we employed Wayne (Venn) analysis to obtain the intersections between up‐regulated DEGs in CM and EC cells treated with CPPs‐Exo, and four other gene lists, which were CREB1 potential target genes in hESC, cancer cells, the CHEA database and the JASPAR database. Finally, we located the *Gpx4* gene. Glutathione peroxidase 4 (GPX4) is an antioxidant enzyme. It is reported as an inhibitor of ferroptosis,^57^ and is the downstream gene in the phenomena of alleviated CM ferroptosis and MI injury caused by cardiac‐specific overexpression of HIP‐55.^58^ In our study, CPPs‐Exo‐derived miR‐27b‐3p down‐regulated the *Sik1* gene, resulting in increased transcriptional activity of CREB1, and then GPX4 was markedly increased. Finally, lipid peroxidation and oxidative stress might be alleviated after MI, leading to cardiac protection after MI, which needs to be further studied.

Primary miRNAs are biosynthesized inside the nucleus via canonical or non‐canonical pathways and then transported to the cytoplasm to be processed into precursor and finally mature elements.^59^ miRNAs contain certain motifs, such as GGAG, UGAG, CCCU, or UCCU that can be recognized by sumoylated heterogeneous nuclear ribonucleoproteins, which are overrepresented in the exosomes and help sorting of miRNAs into exosomes.^60^ The upstream regulatory mechanisms governing exosomal miRNAs are multifarious. They might be up‐regulated by the contribution of myocardium infraction after hypoxic injury, as indicated in the case of miR‐125b‐5p, whose expression levels could also be suppressed by up‐regulation of Smad7.^61^ Another research found significant up‐regulation of miR‐133a‐3p in exosomes could be achieved by overexpression of migration inhibitory factor.^62^ In our case, the upstream mechanism regulating exosomal miR‐27b‐3p requires further study.

There are some limitations in our study. Although, we found that CPPs‐Exo‐derived miR‐27b‐3p and its target gene Sik1 are crucial for the reparative function of CPPs, we do not claim that they are solely responsible for the improvements in cardiac functions brought by CPPs, as other mechanisms could not be excluded. For example, those initiated by proteins or other contents in exosomes. Therefore, other kinds of cardioprotective or angiogenic components need to be investigated. Moreover, increased neovascularization was observed in the CPPs groups; however, additional analysis is needed to determine whether this is sufficient to restore physiological blood flow, since robust arterial input is important for permanent functional improvement. Third, studies using CPPs targeting lung diseases, such as acute lung injury, pulmonary hypertension, are still needed aiming to discover more of their regenerative power, as CPPs could also differentiate into cell lineages of lung, for instance, EC in proximal vessel, pericytes, smooth muscle cells in airway, pulmonary artery, and vein.^17^

Even though our results show CPPs are therapeutic in small rodent models, there are still some challenges associated with utilization of CPPs for clinical applications on cardiac repair, for example, the risk of embolization, immunogenicity, and tumour formation, as well as optimizations on the numbers of cells, the route of injection, the frequency and the best timing for transplantation. CPPs‐Exo would provide a more feasible approach for bench‐to‐bedside translation compared with CPPs. However, some challenges are still there, for instance, no method or specific marker is available to differentiate between exosomes and small microvesicles, which hampers the biological characterization and therapeutic standardization.^63^ To summarize, the therapeutic strategy to use CPPs or CPPs‐Exo holds great promises but warrants further exploration.

In summary, our data indicate that CPPs harbour the unique capability to differentiate into both heart and lung lineages and restore heart functions after injury. Furthermore, the improvement in heart function after injury by CPPs is mediated by CPPs‐Exo, and partially through the miR‐27b‐3p‐SIK1‐CREB1 axis. These findings not only facilitate the understanding of new roles of CPPs in ischemic heart disease but also offer a perspective for developing innovative therapeutic strategies.

## AUTHOR CONTRIBUTIONS

Ying‐Ying Xiao and Xi‐Yong Yu conceived studies, designed experiments, interpreted results and wrote the article. Zhe‐Sheng Chen co‐mentored the postdoc and revised the article. Ying‐Ying Xiao and Luo‐Xing Xia isolated CPPs and CPPs‐Exo, and analysed data. Wen‐Jing Jiang and Jian‐Feng Qin constructed the MI mouse model, did the echocardiography and immunohistochemistry. Li‐Xin Zhao and Ke‐Xin Li did the cell culture, transfection, immunofluorescence, Western blot and RT‐qPCR. Zhan Li and Li‐Juan Huang performed the dual luciferase reporter assay, tube formation assay and ChIP‐qPCR. Xue‐Yan Jiang, Li Wei, and Peng‐Jiu Yu provided technical assistance with experiments.

## FUNDING INFORMATION

This work was supported by the National Key Research and Development Program of China (2022YFE0209700 to Xi‐Yong Yu); Guangdong Provincial Drug Administration Science and Technology Innovation Project – Key Technology Research on Chest Disease Prevention and Treatment Drug Research and Clinical Evaluation (2022ZDZ10 to Xi‐Yong Yu); the fellowship of China Postdoctoral Science Foundation (2022M710890 to Ying‐Ying Xiao); the Epigenetic Drug R&D and Cultivation Plan of the School of Pharmacy, Guangzhou Medical University (06‐410‐2107211 to Ying‐Ying Xiao); the National Natural Science Foundation of China (81900284 to Xue‐Yan Jiang); and the Natural Science Foundation of Guangdong Province (2019A1515011300 to Xue‐Yan Jiang).

## CONFLICT OF INTEREST STATEMENT

The authors declare no competing interests.

## Supporting information


**FIGURE S1.** Identification of CPPs on the heart region and in CPPs we cultured. (A) Representative images of immunofluorecent staining on the heart region of frozen section from mouse E9.5 embryos. Scale bar, 500 μm. (B) The mRNA expression levels of three CPPs markers *Isl1*, *Wnt2*, and *Gli1* in our cultured CPPs at passages 2 and 6. Data are shown as mean ± SEM.; *n* = 3 biological replicates per group; ***p < 0.001 (*t*‐test).

**FIGURE S2.** Determination of the composition in ABC medium. (A) Heat map showing the OD 450 values of CPPs cultured with different concentrations of A83‐01, bFGF and CHIR99021. Data are shown as mean ± SEM; *n* = 3 biological replicates per group. (B) The histogram showing the OD 450 values of CPPs cultured with E8 medium or ABC medium (DMEM/F12 supplemented with 2% B‐27 without vitamin A, 2 mM L‐glutamine, 1% nonessential amino‐acids, 0.1 mM beta‐mercaptoethanol, 1% penicillin/streptomycin, and freshly added A83‐01, bFGF and CHIR99021 to the final concentration of 1 μM, 50 ng/mL, and 12 μM, respectively), supplemented without or with 1.25%, 2.5%, or 5% human platelet lysate (HPL). Data are shown as mean ± SEM; *n* = 6 biological replicates per group; ***p < 0.001 (*t*‐test). (C) Representative images of CPPs stained with Ki67 antibody after cultured in ABC medium with or without HPL. Scale bar, 100 μm.

**FIGURE S3.** The differentiation programs for CPPs. (A) The 12 days program for CPPs to differentiate into fibroblasts (FB) with 10 ng/mL bFGF supplemented in basal medium. (B) The 6 days program for CPPs to differentiate into smooth muscular cells (SMC) with 5 ng/mL TGFβ supplemented in basal medium at the first 2 days, and 10 ng/mL bFGF for the rest 4 days. (C) The 6 days program for CPPs to differentiate into endothelial cells (EC) with Clonetics Endothelial Cell Medium (Lonza, CC‐3156). (D) The 12 days program for CPPs to differentiate into myocardial cells (CM) with 5 μM IWP2, 5 μM purmorphamine, and 5 μM SB431542 supplemented in basal medium at the first 2 days, and only the basal medium for the rest 10 days. (E) The 12 days program for CPPs to differentiate into type II alveolar epithelial cells (AEII) with the first 2 days of EB formation in the EB medium (DMEM/F12 supplemented with 20% knockout serum replacement, 1% nonessential amino acids, 1% penicillin/streptomycin, 1% insulin‐transferrin‐selenite, 0.1 mM beta‐mercaptoethanol, 10 μM of Y‐27632 and freshly added bFGF to 100 ng/mL), and 20 ng/mL Wnt3a, 5 ng/mL FGF‐10, and 5 ng/mL KGF in the basal medium (RPMI 1640 supplemented with 2% B‐27 without insulin, 2 mM L‐glutamine, 1% nonessential amino acids, 1% penicillin/streptomycin and 0.1 mM beta‐mercaptoethanol) for the rest 10 days.

**FIGURE S4.** Extraction and identification of exosomes derived from CPPs (CPPs‐Exo) and analysis of miRNAs in CPPs‐Exo. (A) Schematic diagram of extraction CPPs‐Exo By ultracentrifugation. (B) Representative images of CPPs‐Exo by transmission electron microscopy, scare bar, 100 nm. (C) Particle size distribution in CPPs‐Exo by nanoparticle tracking analysis. Scale bar, 100 nm. (D) Identification of exosome markers, CD63, Alix and Hsp70, in CPPs‐Exo by Western blot. (E) TOP30 miRNAs in CPPs‐Exo by miRNA sequencing. (F) GO analysis of the predicted target genes for miRNAs in CPPs‐Exo after miRNA sequencing. (G) KEGG analysis of the predicted target genes for miRNAs in CPPs‐Exo after miRNA sequencing.

**FIGURE S5.** Evaluation of the risk for forming teratoma of CPPs in the nude mice. (A) The table showing the number of mice with teratoma in two groups, which are five mice injected by mESC and five mice injected by CPPs. (B) Representative photos showing the mice after 90 days of injection with mESC or CPPs. (C) Representative photos of karyotyping analysis in CPPs.

**FIGURE S6.** Detection of Y chromosomes in female MI mice treated with CPPs. (A–E) Representative images of heart sections stained with cTnT antibody and Y‐chromosome probe while counterstaining with DAPI in five different groups that were SHAM (A), MI mice injected by PBS (B), MI injected by CPPs (C), MI injected by CPPs treated with GW4869 (D), and male mouse as the positive control (E). Scale bar, 20 μm.

**TABLE S1.** Details of the antibodies used in the experiment.
**TABLE S2.** Details of the primers used in the experiment.

## Data Availability

All RNA‐seq data reported in this article have been deposited into the NCBI Gene Expression Omnibus database. The accession number for the miRNA‐seq data is GSE231987. The accession number for the scRNA‐seq data is GSE231986. The accession numbers for the bulk‐RNA sequencing data are GSE232665 and GSE232989. All bioinformatics analyses were performed using the following previously published packages with publicly available codes: Cell Ranger Single‐Cell software (https://support.10xgenomics.com/single‐cell‐gene‐expression/software/pipelines/latest/what‐is‐cell‐ranger); Seurat (https://satijalab.org/seurat/); slingshot (https://bioconductor.org/packages/devel/bioc/vignettes/slingshot/inst/doc/vignette.html); and GSEA Broad Institute (https://software.broadinstitute.org/gsea/index.jsp); CytoTRACE (https://cytotrace.stanford.edu/). The datasets supporting conclusions of this article are available from the corresponding authors upon reasonable request.
