# Supplementary figures and images for: Cardiopulmonary progenitors facilitate cardiac repair via exosomal transfer of miR‐27b‐3p targeting the SIK1‐CREB1 axis
Source: Cell Prolif. 2024 Jan 7;57(5):e13593. doi: 10.1111/cpr.13593 (PMC11056695; doi:10.1111/cpr.13593)

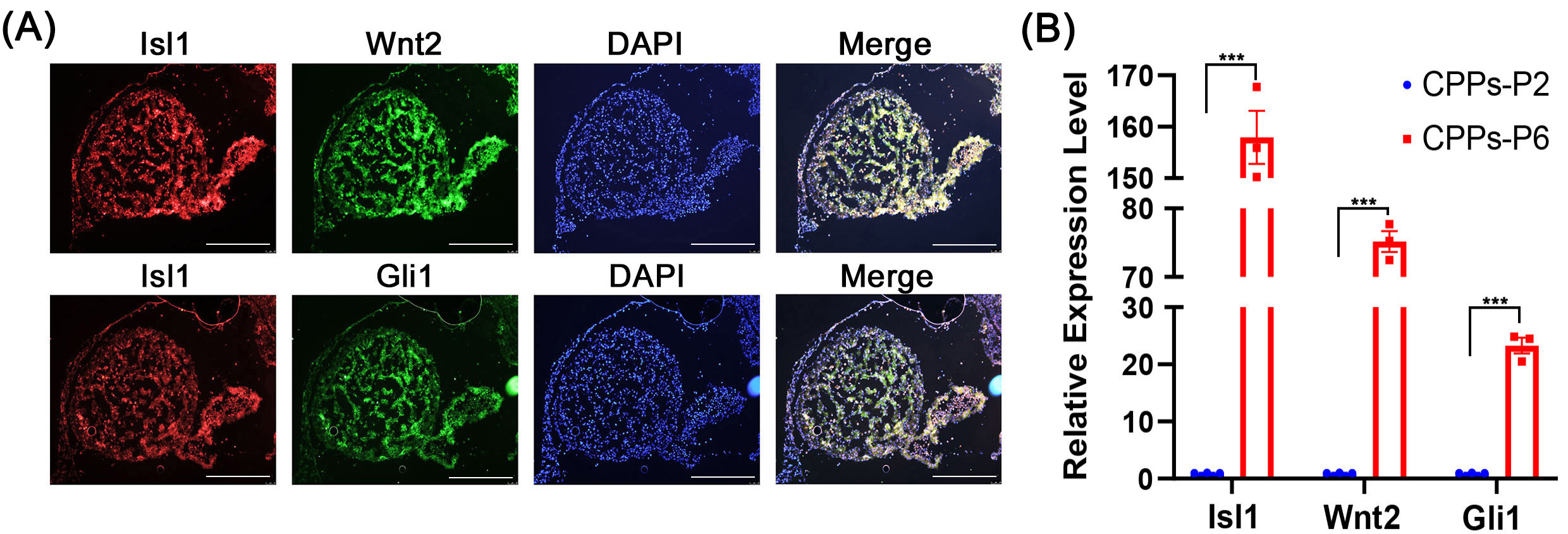

Supplement: Supplementary file 1 — FIGURE S1. Identification of CPPs on the heart region and in CPPs we cultured. (A) Representative images of immunofluorecent staining on the heart region of frozen section from mouse E9.5 embryos. Scale bar, 500 μm. (B) The mRNA expression levels of three CPPs markers Isl1, Wnt2, and Gli1 in our cultured CPPs at passages 2 and 6. Data are shown as mean ± SEM.; n = 3 biological replicates per group; ***p < 0.001 (t‐test). [file CPR-57-e13593-s006.jpg]

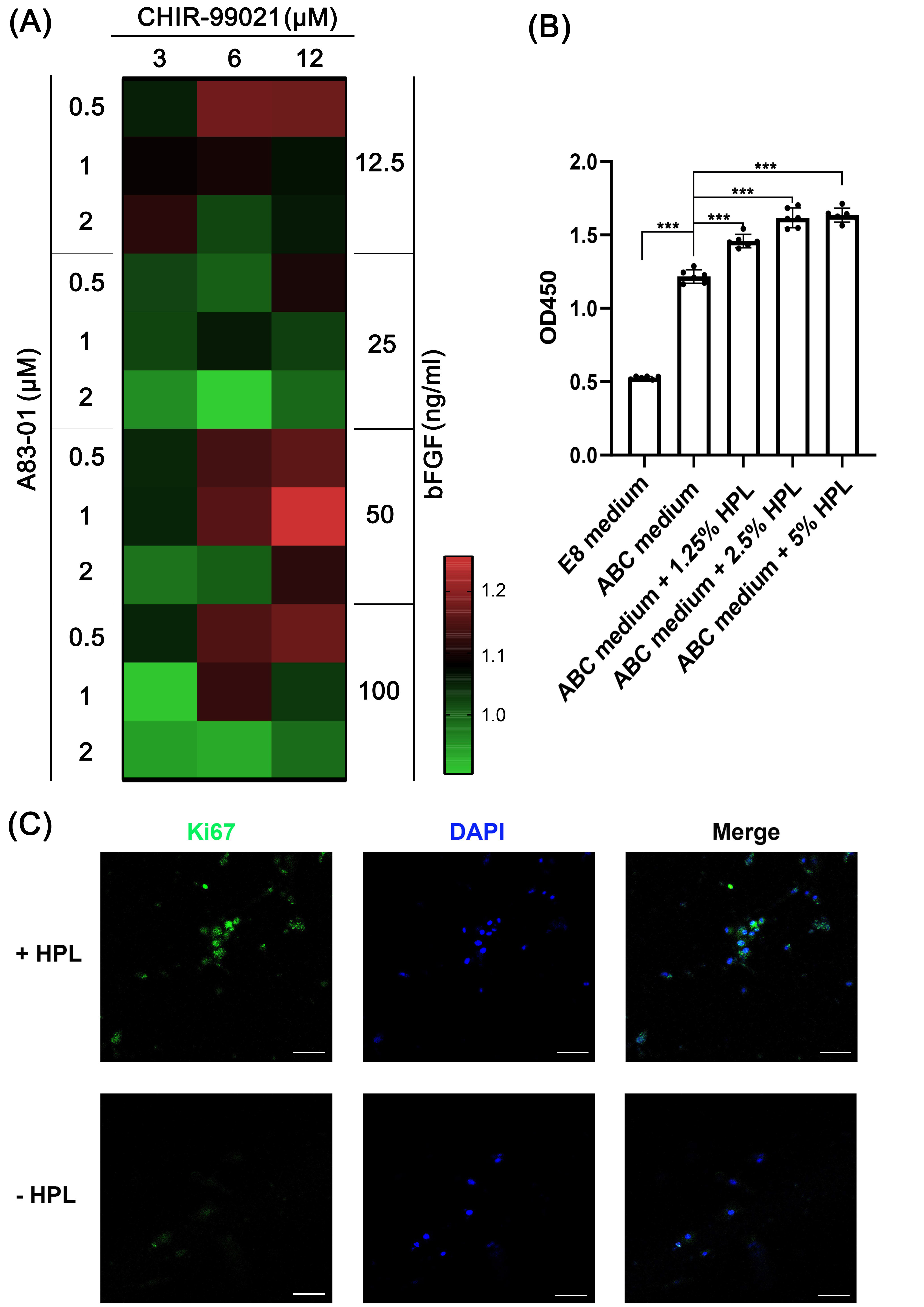

Supplement: Supplementary file 2 — FIGURE S2. Determination of the composition in ABC medium. (A) Heat map showing the OD 450 values of CPPs cultured with different concentrations of A83‐01, bFGF and CHIR99021. Data are shown as mean ± SEM; n = 3 biological replicates per group. (B) The histogram showing the OD 450 values of CPPs cultured with E8 medium or ABC medium (DMEM/F12 supplemented with 2% B‐27 without vitamin A, 2 mM L‐glutamine, 1% nonessential amino‐acids, 0.1 mM beta‐mercaptoethanol, 1% penicillin/streptomycin, and freshly added A83‐01, bFGF and CHIR99021 to the final concentration of 1 μM, 50 ng/mL, and 12 μM, respectively), supplemented without or with 1.25%, 2.5%, or 5% human platelet lysate (HPL). Data are shown as mean ± SEM; n = 6 biological replicates per group; ***p < 0.001 (t‐test). (C) Representative images of CPPs stained with Ki67 antibody after cultured in ABC medium with or without HPL. Scale bar, 100 μm. [file CPR-57-e13593-s005.jpg]

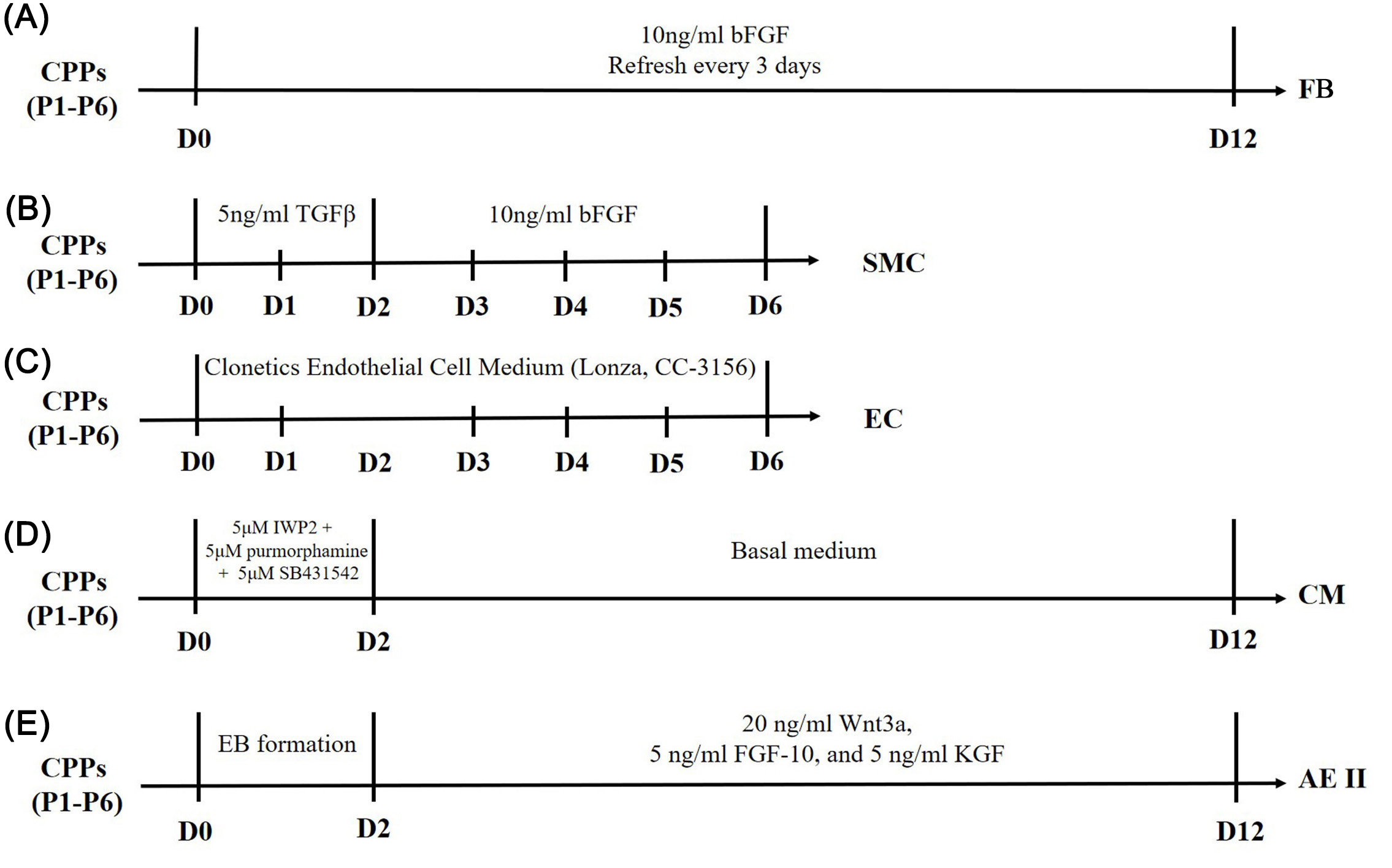

Supplement: Supplementary file 3 — FIGURE S3. The differentiation programs for CPPs. (A) The 12 days program for CPPs to differentiate into fibroblasts (FB) with 10 ng/mL bFGF supplemented in basal medium. (B) The 6 days program for CPPs to differentiate into smooth muscular cells (SMC) with 5 ng/mL TGFβ supplemented in basal medium at the first 2 days, and 10 ng/mL bFGF for the rest 4 days. (C) The 6 days program for CPPs to differentiate into endothelial cells (EC) with Clonetics Endothelial Cell Medium (Lonza, CC‐3156). (D) The 12 days program for CPPs to differentiate into myocardial cells (CM) with 5 μM IWP2, 5 μM purmorphamine, and 5 μM SB431542 supplemented in basal medium at the first 2 days, and only the basal medium for the rest 10 days. (E) The 12 days program for CPPs to differentiate into type II alveolar epithelial cells (AEII) with the first 2 days of EB formation in the EB medium (DMEM/F12 supplemented with 20% knockout serum replacement, 1% nonessential amino acids, 1% penicillin/streptomycin, 1% insulin‐transferrin‐selenite, 0.1 mM beta‐mercaptoethanol, 10 μM of Y‐27632 and freshly added bFGF to 100 ng/mL), and 20 ng/mL Wnt3a, 5 ng/mL FGF‐10, and 5 ng/mL KGF in the basal medium (RPMI 1640 supplemented with 2% B‐27 without insulin, 2 mM L‐glutamine, 1% nonessential amino acids, 1% penicillin/streptomycin and 0.1 mM beta‐mercaptoethanol) for the rest 10 days. [file CPR-57-e13593-s004.jpg]

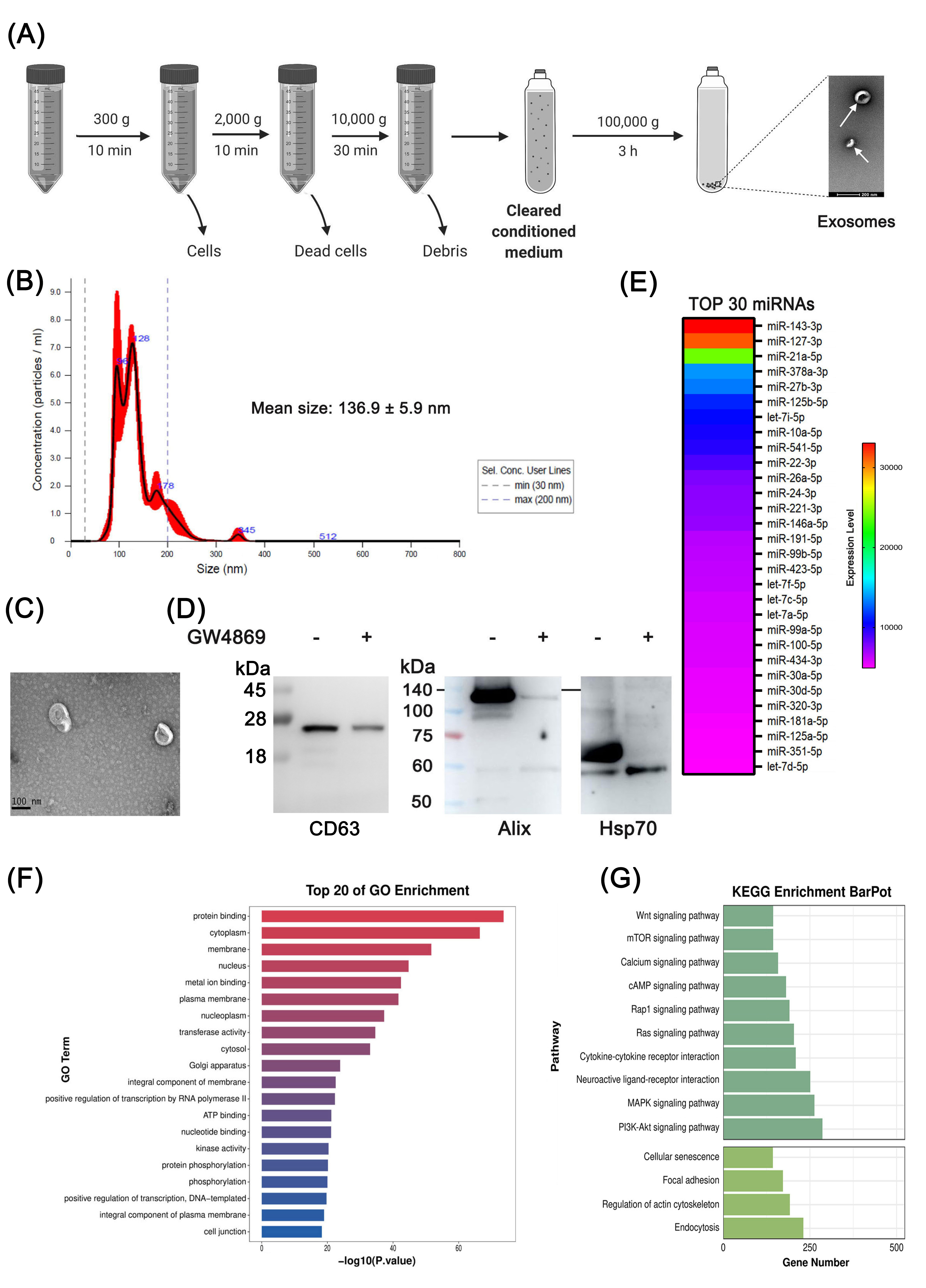

Supplement: Supplementary file 4 — FIGURE S4. Extraction and identification of exosomes derived from CPPs (CPPs‐Exo) and analysis of miRNAs in CPPs‐Exo. (A) Schematic diagram of extraction CPPs‐Exo By ultracentrifugation. (B) Representative images of CPPs‐Exo by transmission electron microscopy, scare bar, 100 nm. (C) Particle size distribution in CPPs‐Exo by nanoparticle tracking analysis. Scale bar, 100 nm. (D) Identification of exosome markers, CD63, Alix and Hsp70, in CPPs‐Exo by Western blot. (E) TOP30 miRNAs in CPPs‐Exo by miRNA sequencing. (F) GO analysis of the predicted target genes for miRNAs in CPPs‐Exo after miRNA sequencing. (G) KEGG analysis of the predicted target genes for miRNAs in CPPs‐Exo after miRNA sequencing. [file CPR-57-e13593-s002.tif]

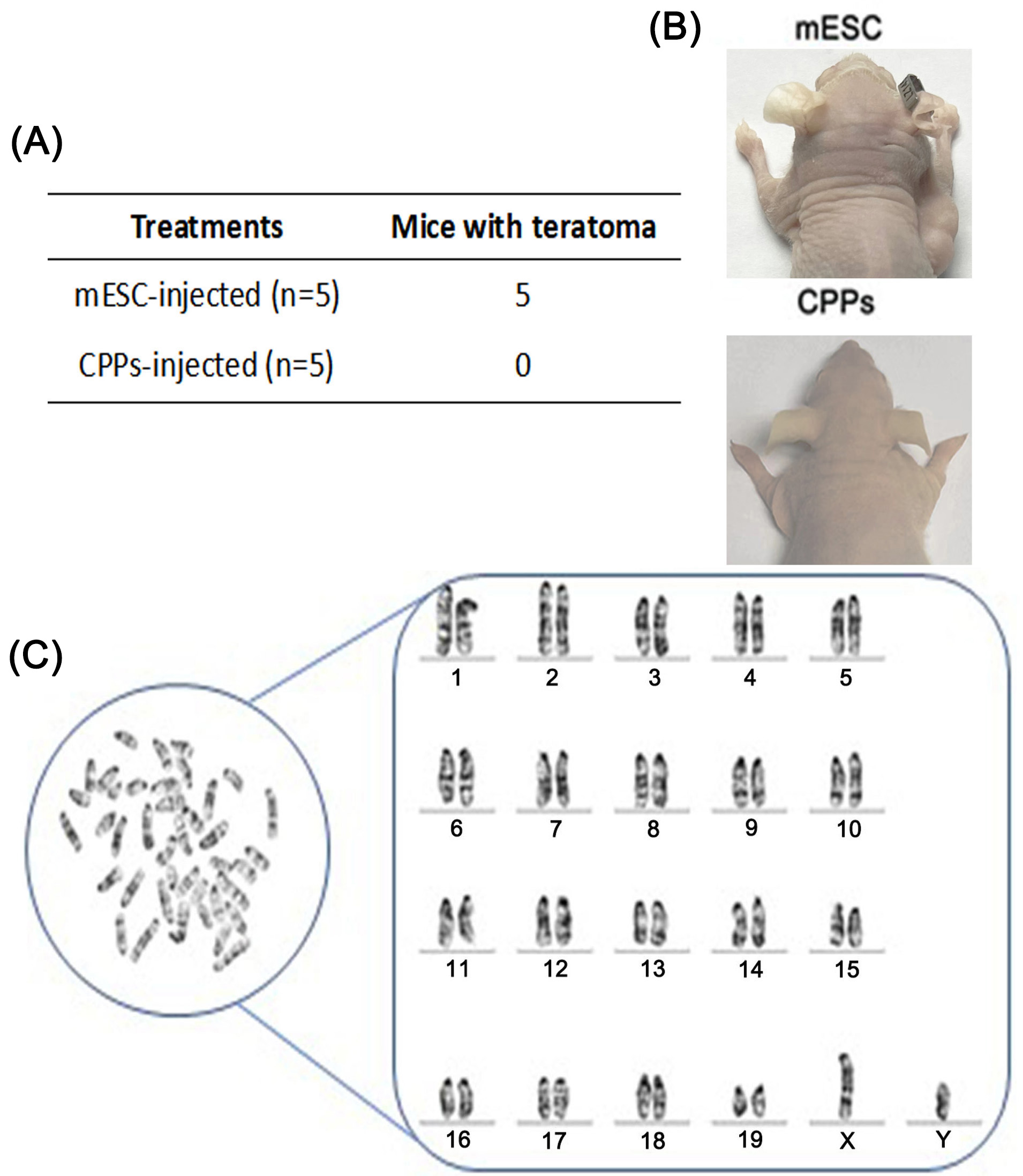

Supplement: Supplementary file 5 — FIGURE S5. Evaluation of the risk for forming teratoma of CPPs in the nude mice. (A) The table showing the number of mice with teratoma in two groups, which are five mice injected by mESC and five mice injected by CPPs. (B) Representative photos showing the mice after 90 days of injection with mESC or CPPs. (C) Representative photos of karyotyping analysis in CPPs. [file CPR-57-e13593-s007.jpg]

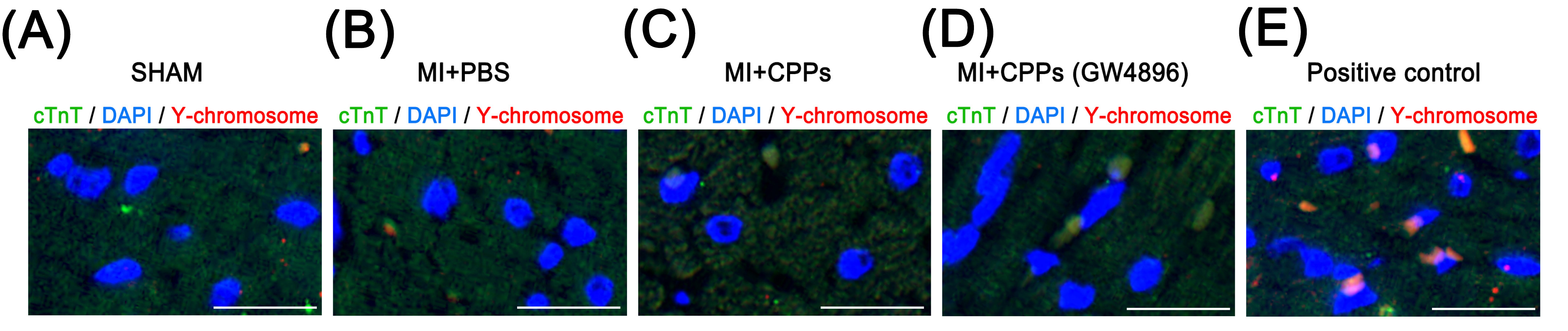

Supplement: Supplementary file 6 — FIGURE S6. Detection of Y chromosomes in female MI mice treated with CPPs. (A–E) Representative images of heart sections stained with cTnT antibody and Y‐chromosome probe while counterstaining with DAPI in five different groups that were SHAM (A), MI mice injected by PBS (B), MI injected by CPPs (C), MI injected by CPPs treated with GW4869 (D), and male mouse as the positive control (E). Scale bar, 20 μm. [file CPR-57-e13593-s001.jpg]
