# Supplementary material for: Cardiopulmonary progenitors facilitate cardiac repair via exosomal transfer of miR‐27b‐3p targeting the SIK1‐CREB1 axis
Source: Cell Prolif. 2024 Jan 7;57(5):e13593. doi: 10.1111/cpr.13593 (PMC11056695; doi:10.1111/cpr.13593)
Supplement: Supplementary file 7 — TABLE S1. Details of the antibodies used in the experiment. TABLE S2. Details of the primers used in the experiment. [file CPR-57-e13593-s003.docx]

**TABLE S1 Details of the antibodies used in the experiment.**

| **Antibody against** | **Manufacturer** | **CAT #** | **Concentration (Application)** |
| --- | --- | --- | --- |
| Isl1 (AF488 Conjugated antibody) | BIOSS | bs-7346R-BF488 | 1:500 (IF); 1:500 (FCM) |
| Wnt2 (AF647 Conjugated antibody) | BIOSS | bs-6133R | 1:500 (IF); 1:500 (FCM) |
| Gli1 （AF405 Conjugated antibody） | Novus | NBP1-78259 | 1:500 (IF); 1:500 (FCM) |
| Isl1 | Thermo Fisher Scientific | MA5-15516 | 1:500 (IF); 1:500 (IHC) |
| Wnt2 | HUABIO | ER1511-4 | 1:500 (IF); 1:500 (IHC) |
| Gli1 | Novus | NB600-600 | 1:500 (IF); 1:500 (IHC) |
| Mesp1 | Novus | NBP1-51613 | 1:500(IF) |
| Ki67 | Abcam | ab16667 | 1:500 (IF); 1:500 (IHC) |
| β-Tubulin | Abcam | ab6046 | 1:1000 (WB) |
| α-SMA | Abcam | ab7817 | 1:500 (IF); 1:500 (IHC) |
| cTnt | Abcam | ab11190 | 1:500 (IF); 1:500 (IHC) |
| Prosurfactant protein C | Abcam | ab90716 | 1:500 (IF) |
| Vimentin | Abcam | ab8069 | 1:500 (IF) |
| CD31 | Abcam | ab28364 | 1:500 (IF) |
| Alexa Fluor® 488 | Abcam | ab150113 | 1:1000 (IF); 1:1000 (IHC) |
| Alexa Fluor® 594 | Abcam | ab150116 | 1:1000 (IF); 1:1000 (IHC) |
| [ALIX](https://www.abcam.com/products/primary-antibodies/alix-antibody-epr23653-32-ab275377.html) | Abcam | ab275377 | 1:1000 (WB) |
| [Hsp70](https://www.abcam.com/products/primary-antibodies/hsp70-antibody-5a5-ab2787.html) | Abcam | ab2787 | 1:1000 (WB) |
| CD63 | Abcam | ab213090 | 1:1000 (WB) |
| [SIK1](https://www.abcam.com/products/primary-antibodies/sik1-antibody-ab217809.html) | Abcam | ab217809 | 1:1000 (WB) |
| CREB | Cell Signaling Technology | 9197T | 1:50 (Chromatin IP) |

**TABLE S2 Details of the primers used in the experiment.**

| **Gene** | **Forward (5' to 3' sequence)** | **Reverse (5' to 3' sequence)** |
| --- | --- | --- |
| *Isl1* (mouse) | AGCAGCAACCCAACGACAAAACTA | GTATCTGGGAGCTGCGAGGACAT |
| *Wnt2* (mouse) | TGTCATGGTGTGAGTGGCTC | CCAGTGCCATCCTGGTTCAT |
| *Gli1* (mouse) | TTGCAGCCAGGAGTTCGATT | GGACTTCCGACAGCCTTCAA |
| *Mesp1* (mouse) | CCCAGGAAAGGCAGGAAATG | GTGCCAAGACCAAAGGAAAAGT |
| *Ssea1* (mouse) | ACATCACCGAGAAGCTGTGG | AGCCAGGGAAGCAGCATTAG |
| *Pdgfra* (mouse) | GTCGGATTTTGGGATCCGGT | GACCTGGCTGTGGGTTTGAG |
| *Mef2c* (mouse) | GGAACACGCCTGTCACCTAA | TTAGCTCTCAAACGCCACA |
| *Kdr* (mouse) | CTACAGACCCGGCCAAACAA | CAGCTTGGATGACCAGCGTA |
| *Nkx2.5* (mouse) | TTCGCCCCCCAAGTGCTCTC | TCCGTCTCGGCTTTGTCCAG |
| *α-Sma* (mouse) | CTTCCAGCCATCTTTCATTGG | GTTCTGGAGGGGCAATGAT |
| *Tnnt2* (mouse) | GCAGCAGAAATACGAAATCAACG | GGCACAGCTTTGACGAGAAC |
| *Tnni3* (mouse) | TTGGATGGGCTGGGCTTTGAA | GCAGAGATCCTCACTCTTCGG |
| *Gata4* (mouse) | AAGACACCCCAATCTCGATATG | GATGCCGTTCATCTTGTGATAG |
| *Pecam* (mouse) | TGAGGAAAGCCAAGGCCAAA | GGCTTCCACACTAGGCTCAG |
| *Myh11* (mouse) | CCTCAAGAGCAAACTCAGGAGA | TCCCTGACATGGTGTCCAATC |
| *Scgb1a1* (mouse) | AACATCATGAAGCTCACGGAGA | AGACACAGGGCAGTGACAAG |
| *Cftr* (mouse) | TTGCCAACTACAGCAGGACA | GAAATCCTTGCACGCTGACC |
| *Sik1* (human) | GTCACTGCAAAACAGCAGCTA | CTTCCTGAGGCACCTCCAAAC |
| *Sik1* (rat) | CGTCTCTCACTCAAGGGCTG | TGGTGCTGTAACTGGAGCAG |
| *Ccnd1* (rat) | TCAGCAGCAGACACCGGA | CTCCTCGCAGACCTCTAGCA |
| *Ccnd2* (rat) | GCTCTGTGTGCTACCGACTT | GGTCCGGATCTTCCACAGAC |
| *Cdc34* (rat) | ATCCTCCATCCCCCAGTTGA | AGGACCTGCTTCCGGATGAT |
| *Cdk4* (rat) | GTCTATGGTCTGGCCCGAAG | TCTCGGAGCAGGGGATCTTA |
| *Cdk6* (rat) | AGGCAAAGACCTCCTTCTGAAAT | TTCAGATGACGAGCTAGCGG |
| *Ccne1* (rat) | CCAGGATAGCAGTCAGCCTT | GCTGAAATGCAGTCTTGGGG |
| *Ccnb1* (rat) | GACAACTGGAGGAAGAGCAGTCA | CATGTACATGGTCTCCTGCAGCA |
| *Gpx4* (human) | TTGGTCGGCTGGACGAGG | GGGACGCGCACATGGT |
| *Gpx4* (rat) | CCGTCTGAGCCGCTTATTGA | CACACGCAACCCCTGTACTT |
| *Gapdh* (mouse) | CGGGTTCCTATAAATACGGACTG | GAAGGGGTCGTTGATGGCAA |
| *Gapdh* (human) | CATGAGAAGTATGACAACAGCCT | AGTCCTTCCACGATACCAAAGT |
| *Gapdh* (rat) | GGCCAAGGTCATCCATGACAAC | ACCAGTGGATGCAGGGATGAT |
| Binding site 1 of *Gpx4* promoter and CREB1 | ATCCTTGCTTCACTGCCTCTG | ATTTCTTCAGAGACAGACTCAGGG |
| Binding site 2 of *Gpx4* promoter and CREB1 | CGCCTTCTTCCCACTCCG | GCTTTCCGCGCCTCCTTT |
| miR-27b-3p | GCCGAGTTCACAGTGGCTAAG | GTCGTATCCAGTGCAGGGTCCGAGGTATTCGCACTGGATACGACGCAGAACT |
| U6 | CTCGCTTCGGCAGCACA | AACGCTTCACGAATTTGCGT |
| universal reversed primer for miRNA qPCR |  | GTCGTATCCAGTGCAGGGTCCG |
